# Research advances in intelligent microenvironment-responsive hydrogels for myocardial infarction therapy

**DOI:** 10.1063/5.0312953

**Published:** 2026-04-02

**Authors:** Jian Li, Jinglin Gao, Kaiyi Zhu, Yuping Gao

**Affiliations:** 1 Third Hospital of Shanxi Medical University, Shanxi Bethune Hospital, Shanxi Academy of Medical Sciences, Tongji Shanxi Hospital, Taiyuan 030032, China; 2 Third Hospital of Shanxi Medical University, Shanxi Bethune Hospital, Shanxi Academy of Medical Sciences, Tongji Shanxi Hospital, Taiyuan 030032, China

## Abstract

Myocardial infarction (MI) poses a severe threat to human life and health. During acute MI, persistent myocardial ischemia and hypoxia induce pathological alterations in the microenvironment. Traditional therapeutic approaches exhibit limited capacity for targeted modulation of this infarcted microenvironment. Consequently, developing therapeutic strategies capable of precisely responding to the pathological microenvironment holds significant importance. Hydrogels, as a class of polymeric biomaterials with excellent biocompatibility, can be engineered into intelligent responsive hydrogels by incorporating environmentally responsive functional groups or constructing intelligent network architectures. These hydrogels are designed to sense and respond to key features of the MI pathological microenvironment, such as temperature, pH, reactive oxygen species, and enzyme concentrations. This review systematically summarizes the design strategies and research advances in intelligent responsive hydrogels for MI therapy over recent years, focusing on their distinct functional capabilities: alleviating oxidative damage, suppressing excessive inflammatory responses, enabling precise drug delivery, and modulating immune activity. Although current research predominantly remains at the preclinical stage and faces numerous challenges, the convergence of materials science and biomedical engineering positions smart responsive hydrogels as promising candidates to deliver innovative solutions for the precise treatment of MI.

## I. INTRODUCTION

Myocardial infarction (MI), representing the most severe clinical manifestation of coronary artery disease, remains one of the leading causes of mortality worldwide.^1,2^ The underlying pathophysiology involves acute coronary occlusion, which results in regional myocardial ischemia and hypoxia.^3^ This disruption of oxygen and nutrient delivery triggers a cascade of biochemical and cellular events that ultimately lead to irreversible cardiomyocyte death.^4^ Current therapeutic strategies include pharmacological treatments, percutaneous coronary intervention (PCI), and coronary artery bypass grafting (CABG).^5^ Although these interventions effectively restore coronary perfusion and improve cardiac performance, they are unable to regenerate lost myocardial tissue.^6^ This limitation maintains metabolic dysfunction within surviving cardiomyocytes, which, in turn, promotes adverse ventricular remodeling and progressive cardiac deterioration.^7,8^ Moreover, conventional pharmacological therapies are limited by poor targeting capability, short retention time, and the complex challenges arising from the dynamic changes of the microenvironment, making it difficult to achieve precise therapeutic regulation.^9^ These limitations highlight the urgent need for innovative therapeutic approaches that preserve viable myocardium, restore cardiac function, and prevent the transition to heart failure.

Recent advances in biomaterials science have introduced new possibilities for the diagnosis and treatment of MI. Emerging biomedical materials, including cardiac patches, hydrogels, nanobiomaterials, and vascular grafts, have shown significant potential in both experimental and clinical settings.^10–12^ In the diagnostic phase, it can monitor abnormal electrocardiogram signals and release drugs on demand, thereby significantly improving the clinical diagnosis and treatment efficiency of MI.^13,14^ In therapeutic contexts, they can serve as delivery platforms for drugs and bioactive molecules that modulate inflammation, regulate immune responses, promote angiogenesis, and suppress post-infarction fibrosis.^15,16^ Collectively, these functionalities provide promising strategies for precise intervention and functional cardiac repair.^17,18^ Among these materials, hydrogels have attracted particular attention in myocardial tissue engineering due to their excellent biocompatibility, tunable mechanical properties, three-dimensional porous structure, and similarity to the native extracellular matrix (ECM).^19,20^ When injected intramyocardially at the infarct site, appropriately engineered hydrogels form *in situ* physical scaffolds that reduce pathological ventricular remodeling and support tissue regeneration.^21^ By restoring the mechanical integrity of the myocardium and enhancing reparative processes, hydrogels contribute to improved cardiac function and better long-term outcomes for patients with MI.^22,23^

Intelligent responsive hydrogels, representing an advanced subclass of hydrogel-based biomaterials, preserve the inherent advantages of conventional hydrogels while integrating specific chemical bonds or functional groups that enable real-time and sensitive responses to dynamic changes in the post-infarction microenvironment.^24–26^ These properties allow precise and controlled release of therapeutic agents, thereby achieving more targeted and effective treatment of MI.^27,28^ During MI progression, the infarcted myocardium undergoes a series of pathological alterations, including tissue ischemia, hypoxia, abnormal reactive oxygen species (ROS) accumulation, pH fluctuations, excessive collagen deposition, and immune-inflammatory responses.^29^ These pathological characteristics not only indicate disease severity but also provide biological cues for the rational design of intelligent responsive hydrogels. By exploiting specific pathological signals such as elevated ROS levels, acidic pH, and overexpression of matrix metalloproteinases (MMPs), these hydrogels can achieve spatiotemporally controlled drug release at the lesion site.^30,31^ This mechanism enhances therapeutic efficacy and minimizes systemic side effects, thereby offering a promising avenue for the development of precision myocardial repair therapies.^32^

This review provides a comprehensive overview of the design principles, recent progress, and therapeutic potential of intelligent responsive hydrogels in the context of MI. Special emphasis is placed on their ability to enable precise, controlled, and adaptive drug delivery in response to pathological cues within the infarcted microenvironment. In addition, the functional advantages and existing challenges that limit the clinical translation of these materials are discussed. Finally, future perspectives and emerging strategies are proposed to overcome these limitations and to promote cardiovascular regeneration and improved clinical outcomes for patients with MI.

**Literature search strategy:** This narrative review was based on a comprehensive literature search of PubMed, Web of Science, Scopus, and Google Scholar for publications from January 2010 to October 2025. Search terms included “myocardial infarction,” “intelligent hydrogel,” “responsive hydrogel,” “stimuli-responsive,” “pH-responsive,” “ROS-responsive,” “MMP-responsive,” “thermoresponsive,” “drug delivery,” and “cardiac repair.” We primarily included original research articles and selected reviews providing critical mechanistic or translational insights. Inclusion criteria were: (1) studies on intelligent responsive hydrogels for MI therapy; (2) English-language publications; (3) peer-reviewed articles with clear material design and therapeutic outcome descriptions. Exclusion criteria included: (1) conventional non-responsive hydrogels; (2) non-MI cardiovascular diseases; (3) conference abstracts or reviews without original data. While not following a systematic review protocol, the selection process was guided by these criteria to ensure comprehensive coverage of representative, high-quality studies, illustrating key design principles and therapeutic strategies in this field.

## II. DESIGN PRINCIPLES OF INTELLIGENT RESPONSIVE HYDROGELS

**FIG. 1.**
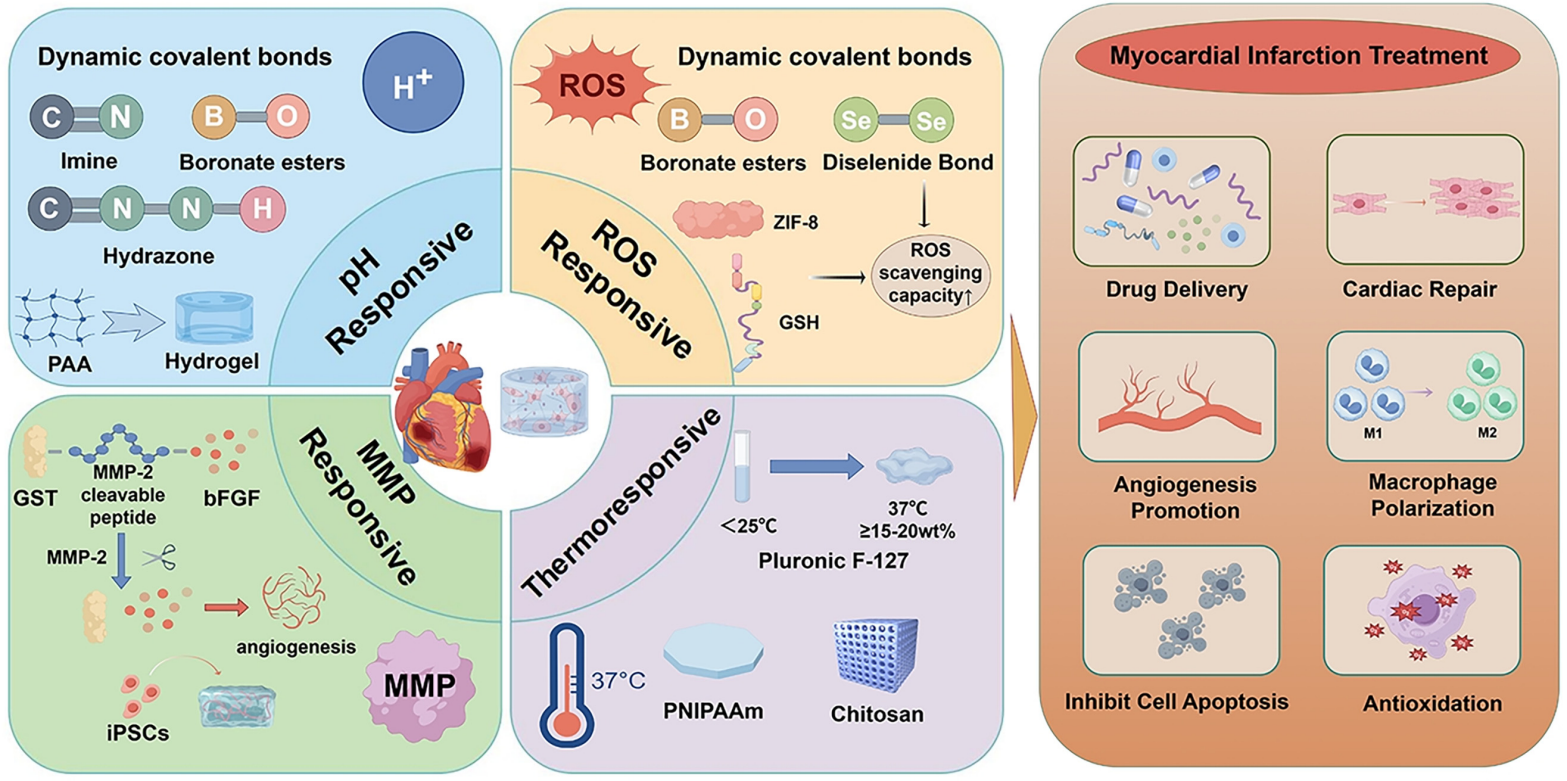
Schematic illustration of the design strategies and therapeutic mechanisms of intelligent microenvironment-responsive hydrogels for MI treatment.

A comprehensive understanding of the dynamic evolution of the pathological microenvironment during MI is fundamental to the rational design of polymer networks that can achieve spatiotemporally controlled release of therapeutic agents. In this context, intelligent responsive hydrogels, which are constructed from functional polymer backbones and cross-linking moieties, exhibit environment-specific disassembly behaviors when exposed to pathological conditions characteristic of MI.^33,34^ For instance, certain dynamic covalent bonds or supramolecular cross-linking structures remain stable under physiological pH but undergo rapid dissociation in acidic microenvironments, thereby enabling the precise release of encapsulated drugs at the infarct site.^35,36^ Similarly, hydrogels that incorporate ROS-responsive groups, such as disulfide bonds or boronate esters, undergo oxidative degradation in the presence of elevated ROS levels.^37^ This process not only allows controlled release of therapeutic payloads but also contributes to local ROS scavenging, thereby exerting dual therapeutic effects.^38,39^ In addition, MMP-responsive hydrogels, which are engineered with MMP-cleavable peptide sequences, are selectively degraded by these enzymes that are overexpressed in the infarcted myocardium.^40,41^ This enzymatic responsiveness induces localized structural changes in the hydrogel network, promoting site-specific accumulation and targeted delivery of bioactive agents.^42^ The following sections provide an in-depth discussion of the pathological features of the MI microenvironment, the fundamental design strategies underlying stimuli-responsive hydrogel systems, and the latest advancements in preclinical and clinical research (Fig. 1). Collectively, these insights aim to inform future directions in the development of intelligent biomaterial-based cardiac therapies.

### A. pH-responsive hydrogels

#### 1. pH dynamics in the MI microenvironment

Under normal physiological conditions, cardiomyocytes primarily rely on aerobic metabolism to generate Adenosine Triphosphate (ATP). However, during MI, coronary artery obstruction induces local myocardial ischemia and hypoxia, forcing cells to shift from aerobic to anaerobic metabolism.^43,44^ This metabolic shift is accompanied by substantial lactate production, which subsequently dissociates into lactate and H^+^.^45^ The rapid accumulation of H^+^ in the ischemic region decreases intracellular pH to approximately 6.0–6.5, thereby creating a locally acidic microenvironment.^46,47^ In addition, impaired ischemic conditions hinder the timely clearance of CO_2_ generated by cardiomyocyte metabolism. CO_2_ reacts with water to form carbonic acid, which further dissociates into H^+^, exacerbating intracellular acidification.^48^ The resulting acidosis triggers a cascade of pathological responses: accumulated H^+^ activates the Na^+^/H^+^ exchanger (NHE), which extrudes H^+^ in exchange for Na^+^ influx. This exchange causes intracellular Na^+^ overload and subsequently drives the Na^+^/Ca^2+^ exchanger (NCX) to operate in the reverse mode, leading to Ca^2+^ influx.^49,50^ The resultant calcium overload activates calcium-dependent proteases, endonucleases, and phospholipases, ultimately promoting apoptosis and necrosis. Furthermore, the acidic environment destabilizes lysosomal membranes, releasing acid hydrolases that further compromise cellular integrity.^51^ These pH alterations not only represent a pathophysiological hallmark of MI but also provide a compelling biochemical rationale for the design of pH-responsive hydrogel systems.

#### 2. Design principles of pH-responsive hydrogels

##### a. pH-sensitive group chemical responsiveness.

**FIG. 2.**
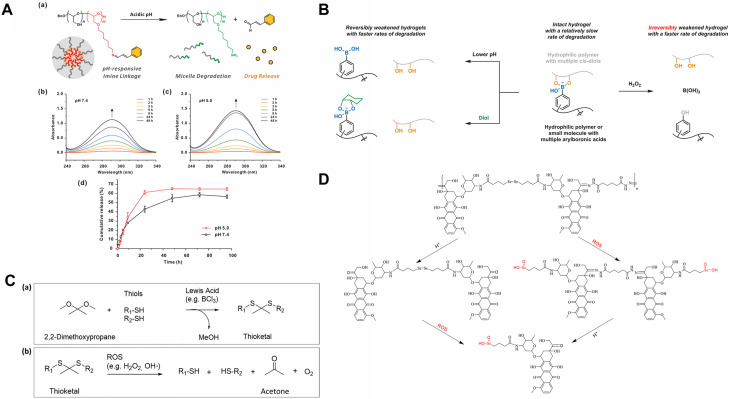
Schematic illustration of the environmentally responsive degradation mechanisms of imine, boronate ester, diselenide, and thioketal linkages. (A) (a) Schematic illustration of the degradation of pH-responsive imine crosslinkers under acidic conditions, resulting in the release of cinnamaldehyde. (b) and (c) UV-visible absorption spectra of the system. (d) Cumulative release profiles of cinnamaldehyde at pH 5.0 and pH 7.4. Reproduced with permission from Han *et al.*, ACS Appl. Bio Mater. **4**, 2465–2474 (2021). Copyright 2021 ACS Publications.^52^ (B) Schematic illustration of the dissolution mechanisms of arylboronate ester-crosslinked hydrogels. The network can be disrupted by lowering the pH, introducing diol-containing molecules, or exposure to ROS. The ROS-triggered cleavage of arylboronate esters is irreversible, whereas the effects induced by pH or diols are reversible. Reproduced with permission from Han *et al.*, J. Mater. Chem. B, **10**, 6263 (2022). Copyright 2022 Royal Society of Chemistry.^53^ (C) (a) TK bonds are formed via the reaction between thiols and ketones. (b) TK bonds can be cleaved by ROS. Reproduced with permission from Rinaldi *et al.*, Polymers **14**, 687 (2022). Licensed under a Creative Commons Attribution (CC BY) license/Copyright 2022 MDPI.^54^ (D) Schematic illustration of the pH/oxidation dual‐triggered degradation of PDOX, leading to the release of selenious acid (DOX‐SeOOH). Reproduced with permission from Hu *et al.*, Molecules **29**, 3837 (2024). Licensed under a Creative Commons Attribution (CC BY) license/Copyright 2024 MDPI.^55^

**FIG. 3.**
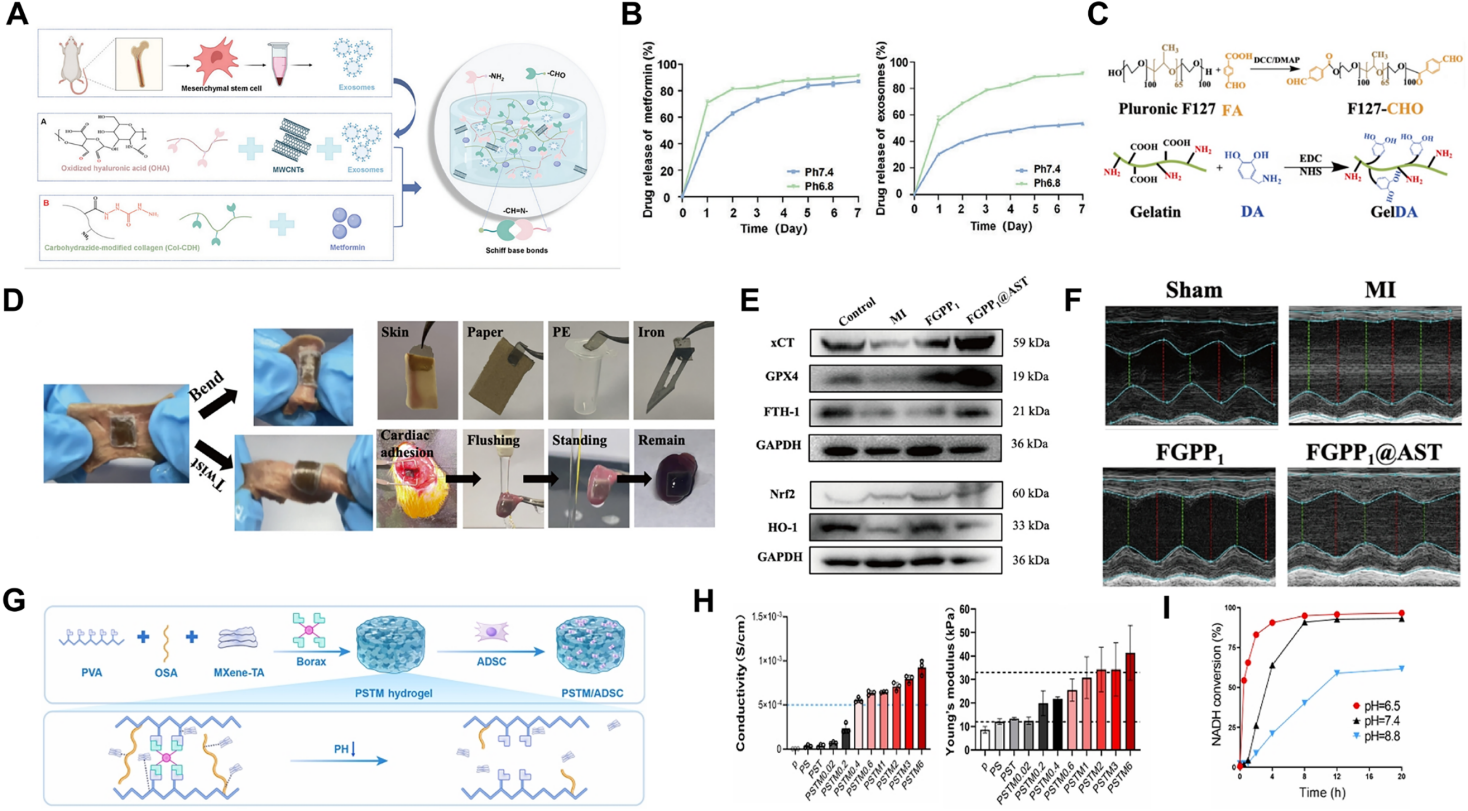
Application of pH-responsive hydrogels in the treatment of MI. (a) Schematic illustration of the preparation and composition of Gel@MSN/miR-21-5p hydrogel. (b) Release profiles of metformin and exosomes from the hydrogel under pH 6.8 and pH 7.4 conditions. Reproduced with permission from Cheng *et al.*, Adv. Sci. **12**, 2410590 (2025). Licensed under a Creative Commons Attribution (CC BY) license/Copyright 2025 Wiley.^61^ (c) The schematic illustrates the formation of F127-CHO-GelDA through a Schiff base reaction between F127-CHO and GelDA. (d) The FGPP1 hydrogel maintained strong adhesion to cardiac tissue even after bending and twisting and exhibited excellent adhesion on various substrates (skin, paper, polyethylene, and iron) as well as *in vivo* cardiac tissue after MI surgery. (e) Compared with the MI group, the FGPP1@AST hydrogel group showed markedly increased expression of ferroptosis-related proteins (xCT, GPX4, and FTH-1) and Nrf2/HO-1 pathway proteins. (f) Representative echocardiographic images of hearts from different groups at two weeks after MI and following treatment. Reproduced with permission from Li *et al.*, J. Controlled Release **384**, 113874 (2025). Copyright 2025 Elsevier.^62^ (g) Schematic illustration of the preparation process of the PSTM/Adipose-Derived Stem Cell (ADSC) hydrogel. (h) Comparison of electrical conductivity and elastic modulus among PS, PST, and PSTM(x) hydrogels. (i) The NADH scavenging activity of the PSTM hydrogel was measured at pH 6.5, 7.4, and 8.8 using a colorimetric assay. Reproduced with permission from Chen *et al.*, Research **8**, 0973 (2025). Licensed under a Creative Commons Attribution (CC BY) license/Copyright 2025 Research.^63^

pH-responsive hydrogels are engineered with acid-labile chemical linkages such as imines, hydrazones, and boronate esters that undergo reversible hydrolysis under acidic conditions, thereby enabling the controlled release of encapsulated therapeutic agents^56–58^ (Table I). Imine bonds, formed by the condensation of an aldehyde and a primary amine group, exhibit dynamic reversibility and pH-tunable stability, making them valuable linkages in the hydrogel design.^59^ They remain stable under physiological pH (7.4), maintaining network integrity and preventing premature drug release. Within the mildly acidic microenvironment of infarcted myocardium, protonation of the imine nitrogen increases its susceptibility to hydrolysis, resulting in C=N bond cleavage and subsequent network degradation [Fig. 2(A-a)]. UV-Vis spectroscopic analysis revealed pronounced spectral changes during imine bond cleavage, where the absorbance intensity at 240–340 nm increased markedly at pH 5.0 compared with pH 7.5 [Figs. 2(A-b)–2(A-c)]. Moreover, pH-dependent release kinetics showed strong sensitivity to acidic conditions, with significantly accelerated drug release at pH 5.5, confirming the pH-responsive degradation mechanism [Fig. 2(A-d)]. This selective, acid-sensitive response underscores their potential for controlled and localized therapy in cardiac repair applications. For instance, Li *et al.* developed an injectable hydrogel, Gel@MSN/miR-21-5p, composed of functional mesoporous silica nanoparticles (MSNs) and microRNA-21-5p encapsulated within a hydrogel matrix.^60^ The matrix is formed from amino-functionalized MSN and aldehyde-terminated polyethylene glycol (PEGCHO). The positively charged MSN binds to miR-21-5p, while the aldehyde groups of PEGCHO react with the amino groups on MSN through imine (Schiff base) cross-linking, resulting in the formation of a stable hydrogel network. According to *in vitro* studies, the sol-to-gel phase transition of this system was completed in about 5 min. When the hydrogel is injected into the MI site, the locally acidic microenvironment breaks the imine bonds, causing degradation of the hydrogel matrix and the consequent release of MSN/miR-21-5p complexes, which promote immunoregulation and angiogenesis. It should be noted that the optimal therapeutic time window of the hydrogel has not yet been clearly defined in this study. Further optimization of the Gel@MSN/miR-21-5p dosage, along with long-term efficacy and safety evaluations, is required to facilitate its eventual translation into clinical applications. Similarly, Cheng *et al.* reported a pH-responsive conductive hydrogel fabricated via Schiff base reactions between oxidized hyaluronic acid (OHA) and carboxylated multi-walled carbon nanotube (MWCNT)-modified collagen (Col-CDH)^61^ [Fig. 3(a)]. When both OHA and Col-CDH were at a concentration of 10%, the gelation time was approximately 20 s, ensuring rapid *in situ* gel formation and good injectability. *In vitro* results showed that both metformin and exosomes exhibited faster release at pH 6.8 than at pH 7.0. Owing to the amino groups in metformin and the acid-sensitive imine bonds in the hydrogel, metformin showed a more pronounced initial release. This reversible linkage enables pH-responsive, intelligent release while maintaining bioactivity and therapeutic efficacy [Fig. 3(b)]. In another study, Li *et al.* designed a conductive and bioadhesive hydrogel through dynamic cross-linking of dopamine-functionalized gelatin (Gel-DA) with cyclodextrin-modified Pluronic F127 (F127-CHO), incorporating polydopamine–polypyrrole (PDAPpy) nanoparticles to enhance electrical conductivity, mechanical strength, and tissue adhesion^62^ [Figs. 3(c) and 3(d)]. The incorporation of PDA–PPy also accelerated the gelation process of the hydrogel, enabling FGPP4 to achieve rapid gelation within 2 min. Thermosensitive F127 micelles were used to encapsulate astragaloside IV (AST), while the pH-responsive Schiff base linkages ensured targeted release within the infarcted myocardium. This system effectively activated the Nrf2/HO-1 signaling pathway, inhibited hypoxia-induced ferroptosis, and improved post-infarction cardiac function [Figs. 3(e) and 3(f)]. The hydrogel showed a sustained-release effect for AST, but the release amount remains difficult to control precisely. Future studies will develop more controllable systems and explore the addition of other active components, such as vascular endothelial growth factor (VEGF) or stem cells, to improve cardiac repair.

**TABLE I.** Microenvironment-responsive chemical bonds: degradation mechanisms, kinetics, and release characteristics.

Responsive chemical bonds	Chemical structure	Stimulus type in MI	Bond dissociation mechanism	Degradation kinetics	Release characteristics	References
Imine (Schiff base)	R_1_-CH=N-R_2_	pH < 6.5	Acid-catalyzed hydrolysis Nucleophilic addition of water to protonated imine	pH > 7.0 t_1/2_ 6–12 h pH < 5.0 t_1/2_ 1–3 h	Sustained pH-responsive release; reversible gelation enables self-healing and adaptability	58 and 59
Boronate ester	Ar-B(OR)_2_B(OR)_3_	pH < 7.4ROS	pH: reversible hydrolysis ROS: irreversible oxidation	pH 6.5–7.0 slow pH < 5.0 faster 1 mM H_2_O_2_ t_1/2_ 5 min 10 mM H_2_O_2_ t_1/2_ 70 s	pH/ROS dual-responsive release Higher physiological stability and specific release ability of the lesion	206–208
Diselenide	R_1_-Se-Se-R_2_	ROS	R_1_-Se-Se-R_2_ + H_2_O_2_ → [R_1_-Se(O)-Se-R_2_] → R_1_-SeOH + R_2_-SeOH; R_1_-SeOH + H_2_O_2_ → R_1_-SeO_2_H + H_2_O	H_2_O_2_ t_1/2_ about 30 min–2 h	Sustained and controlled ROS-responsive irreversible release	209 and 210
Thioketal	R_1_-S-C(CH_3_)_2_-S-R_2_	ROS	ROS oxidation of sulfur oxides Hydrolysis and cleavage of the C–S bond	H_2_O_2_ t_1/2_ 11–20 h	ROS-responsive release ROS scavenger	211 and 212

Boronate ester bonds represent a class of multifunctional, stimuli-responsive linkages that show dual sensitivity to pH and ROS.^56^ This unique feature makes them particularly well suited for addressing the complex pathological microenvironment of MI, which is characterized by acidosis and oxidative stress. These dynamic B–O bonds are formed through the reversible condensation of boronic acids with cis-diol compounds and can undergo cleavage by hydrolysis under acidic conditions or by oxidation in the presence of ROS. Boronate-based hydrogels can be designed as either reversible or irreversible crosslinked networks, each showing distinct degradation behaviors in response to environmental stimuli [Fig. 2(B)]. Their dual responsiveness to pH and ROS enables controlled drug release, allowing a rapid burst of antioxidants during acute oxidative stress followed by sustained release as tissue recovery progresses. In addition, the reversible nature of boronate linkages confers self-healing ability and good biocompatibility, since the resulting degradation products exhibit low cytotoxicity and can be readily metabolized and eliminated. For instance, Luo *et al.* fabricated a hydrogel using 2-formylphenylboronic acid (2-FPBA) as a crosslinker, forming Schiff bases with quaternized carboxymethyl chitosan (QCMCS) and boronate esters with tannic acid (TA).^64^ Elevated ROS in ischemic regions cleaved boronate bonds, while acidic pH destabilized both linkages, triggering the release of S1QEL1.1 and FT011 to attenuate myocardial injury. Chen *et al.* designed a multifunctional hydrogel integrating metabolic, electrical, and mechanical regulation.^63^ The PVA/OSA/borax/tannic acid hydrogel (PSTM) was crosslinked through boronate ester bonds, enabling adaptive mechanical strength and conductivity under acidic conditions [Figs. 3(g) and 3(h)]. At pH 6.5, the hydrogel exhibited the highest NADH scavenging activity, confirming that the borate ester bonds in the system respond to the acidic environment, triggering the release of embedded MXene-TA nanozymes to catalyze the oxidation of NADH to NAD^+^ [Fig. 3(i)]. This process improved myocardial energy metabolism, enhanced stem cell viability and electrical coupling, and ultimately restored cardiac function after injury.

##### b. pH-responsive materials.

pH-responsive materials, a specialized subclass of stimuli-responsive polymers, can dynamically modulate their physicochemical properties in response to variations in environmental pH.^65^ This adaptive behavior provides distinct advantages for the drug delivery system design, enabling site-specific and temporally controlled release of therapeutic agents. By leveraging the pH gradients that exist between healthy and pathological tissues or among intracellular compartments, these materials facilitate targeted drug delivery with enhanced bioavailability and reduced off-target effects, thereby improving therapeutic efficacy while minimizing systemic toxicity.^66,67^

Poly(acrylic acid) (PAA), synthesized through the polymerization of acrylic acid monomers, is one of the most extensively studied pH-responsive polymers because of its high density of ionizable carboxyl groups (–COOH) along the polymer backbone.^68^ These functional groups undergo reversible protonation and deprotonation in response to environmental pH fluctuations, which underpin the pH sensitivity of PAA. With an intrinsic pKa of approximately 4.25, PAA remains predominantly protonated under acidic conditions (pH < 4.25) and becomes progressively deprotonated as the pH exceeds this threshold. Within the pH range of 4.0–6.4, deprotonation of carboxyl groups induces electrostatic repulsion among polymer chains, leading to network expansion and increased hydrogel swelling.^69^ At higher pH levels, counterions partially screen these repulsive interactions, thereby reducing the swelling capacity.^70^ This responsive swelling behavior enables PAA-based hydrogels to dynamically regulate their physical state and to achieve controlled release of encapsulated agents, demonstrating significant potential for MI therapy. Garbern *et al.* developed a dual pH and temperature-responsive injectable hydrogel based on the terpolymer p(NIPAAm-co-PAA-co-BA), composed of N-isopropylacrylamide (NIPAM), acrylic acid (AA), and butyl acrylate (BA).^71^ In this system, NIPAM imparts thermosensitivity, whereas PAA introduces pH responsiveness, enabling the hydrogel to transform from a sol to a gel state at 37 °C under acidic conditions characteristic of ischemic myocardial tissue. This property facilitates localized and sustained delivery of basic fibroblast growth factor (bFGF), thereby promoting angiogenesis and cardiac repair. At 28 days post-surgery, a chronic inflammatory response was observed near the polymer injection site, warranting further investigation into its duration and potential effects on angiogenesis and cardiac function. Future studies should strengthen safety evaluations, including assessments of potential side effects, toxicity, and long-term complications. In another study, Shahid *et al.* fabricated a pH-sensitive nanocomposite hydrogel (NCH) for ticagrelor (TG) delivery via free-radical polymerization of chitosan (CH) and AA, using N,N′-methylenebisacrylamide (MBAA) as the crosslinker and potassium persulfate (KPS) as the initiator, with incorporation of thiolated chitosan (TCH)-based nanoparticles.^72^ The resulting NCH exhibited pH-dependent drug release, improved bioavailability, an extended half-life, and an increased area under the plasma concentration–time curve (AUC). By reducing dosing frequency and effectively inhibiting platelet aggregation, this system represents a promising therapeutic platform for MI management with enhanced patient compliance. It is worth noting that this study mainly examined the *in vitro* and *in vivo* release behavior of NCHs, but their stability requires further investigation. Although the prepared NCHs showed pH-responsive properties, they lacked targeting ability. Future research should consider linking NCHs with targeting ligands to improve delivery efficiency at specific sites.

These studies indicate that pH-responsive hydrogels can effectively utilize the acidic microenvironment of infarcted myocardium to achieve controlled drug release and targeted cardiac repair. By combining tunable chemical linkages with bioactive components, these materials promote angiogenesis, regulate inflammation, and improve cardiac function after myocardial infarction. However, regional differences in myocardial pH may lead to variations in degradation behavior and drug release profiles *in vivo*, highlighting the need for more precise spatiotemporal control. Furthermore, the structural stability, reproducibility, and scalability of multifunctional hydrogels require further optimization to meet clinical translation requirements. Future research should emphasize hydrogels with multiple stimuli responsiveness and feedback-regulated release mechanisms, integrating 3D bioprinting and injectable microgel technologies for controllable cardiac repair. Developing multi-level pH-sensitive systems with diverse pKa linkages will better match infarct evolution. pH gradient microfluidics, isotope tracking, and cardiac co-culture models can elucidate structure–function correlations, providing optimization strategies that advance these materials from bench to bedside for MI treatment.

### B. ROS-responsive hydrogels

#### 1. ROS dynamics in the MI microenvironment

ROS is single-electron reduction products of oxygen *in vivo*, primarily including superoxide anion (O_2_^−^), hydrogen peroxide (H_2_O_2_), and hydroxyl radical (• OH).^73^ Their dynamic fluctuations are closely associated with pathological processes in the body. At low or physiological levels, ROS acts as signaling molecules participating in intracellular signal transduction within cardiac cells, regulating physiological activities such as cardiomyocyte growth, proliferation, and differentiation.^74^ However, during MI, particularly following myocardial reperfusion, ROS concentrations in ischemic myocardium surge to levels tens of times higher than normal, inducing oxidative stress, disrupting cell membranes, and ultimately leading to cell death.^75,76^

Under cardiac physiological conditions, ROS generation mainly originates from the mitochondrial electron transport system, xanthine oxidase-catalyzed reactions, NADPH oxidase (NOX)-mediated oxidation processes, and nitric oxide (NO) synthase metabolic activity.^77^ During MI, ischemia impedes mitochondrial oxidative phosphorylation, increasing electron leakage from complexes I and III of the electron transport chain, directly generating O_2_^−^.^78^ In the process of myocardial I/R, xanthine oxidase activity is significantly enhanced, catalyzing the oxidation of hypoxanthine and xanthine to produce O_2_^−^ and H_2_O_2_, becoming a major source of ROS.^79^ Additionally, infiltrating neutrophils and macrophages in the infarct zone generate substantial ROS through NOX catalysis.^80^ In certain cases, endothelial nitric oxide synthase undergoes uncoupling, producing O_2_^−^ instead of NO. Excessive ROS exhibits potent oxidative activity, attacking biological macromolecules such as cell membranes, proteins, and nucleic acids, leading to cardiomyocyte injury and death, thereby exacerbating myocardial tissue damage.^73^ Therefore, antioxidant therapeutic strategies targeting ROS, such as ROS-responsive hydrogels, hold significant clinical importance in the treatment of MI.

#### 2. Design principles of ROS-responsive hydrogels

ROS-responsive hydrogels designed for MI therapy can generally be classified into three major design strategies. (1) Bond-based systems, which employ ROS-labile linkages such as diselenides, boronate esters, or thioacetals that undergo oxidative cleavage; (2) Material-based systems, which rely on the intrinsic physicochemical transformations of ROS-sensitive polymers; and (3) Composite systems, which incorporate exogenous ROS scavengers to enhance antioxidant capacity. These strategies share a common goal of targeted drug delivery and attenuation of oxidative stress.

##### a. ROS-sensitive chemical bonds.

**FIG. 4.**
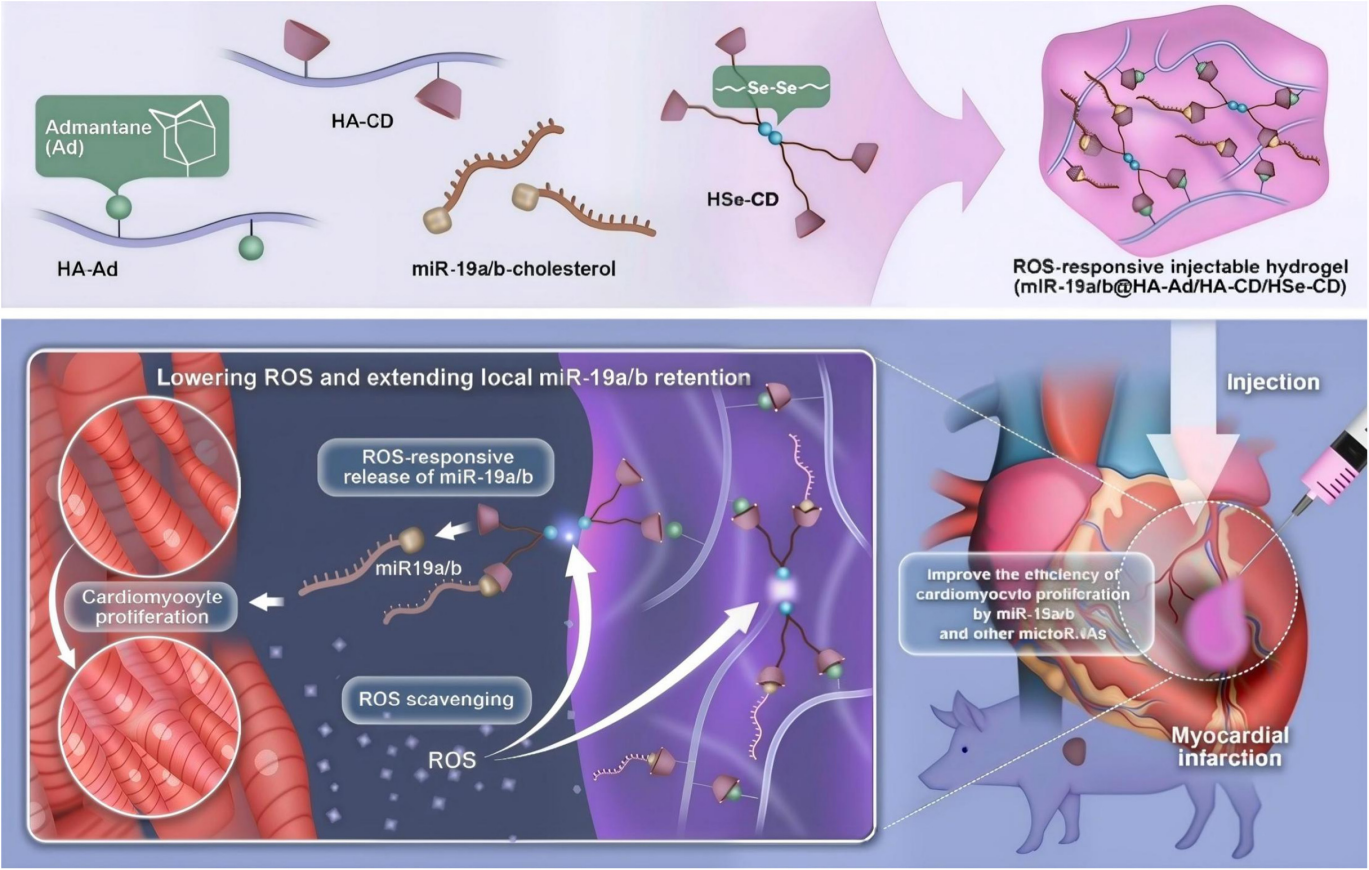
Schematic illustration of the synthesis of miR‐19a/b@HA‐Ad/HA‐CD/HSe‐CD hydrogel and its injection into the infarcted region of a porcine heart. The hydrogel achieves ROS‐responsive sustained release of miR‐19a/b via diselenide bonds, effectively promoting cardiomyocyte proliferation, scavenging excessive ROS in the MI microenvironment, and thereby improving inflammation and cardiac function. Reproduced with permission from Wang *et al.*, Biomaterials **312**, 122732 (2025). Copyright 2025 Elsevier.^85^

**FIG. 5.**
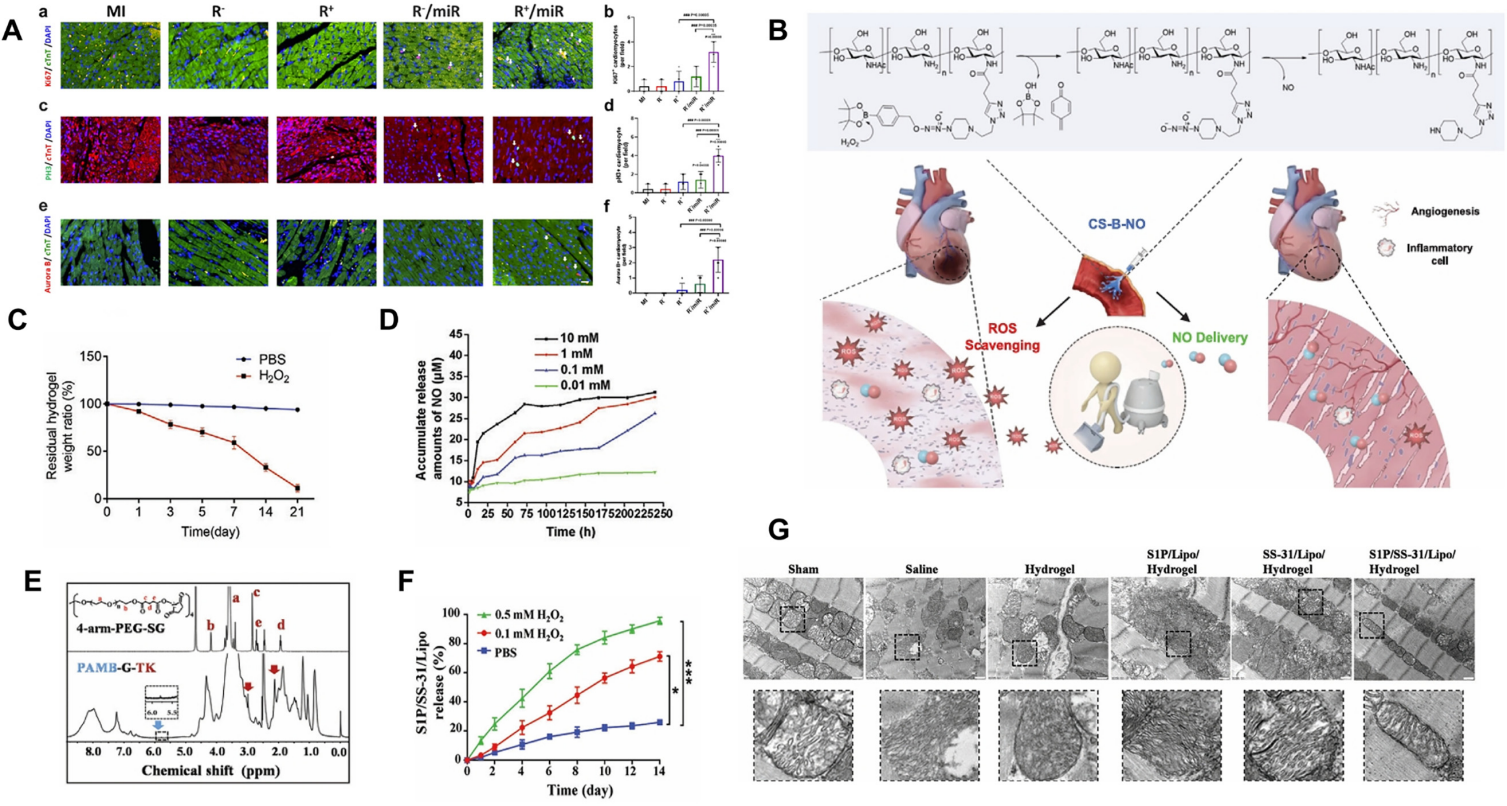
Application of ROS-responsive hydrogels in the treatment of MI. (a) Representative images and quantitative analysis of Ki67, pH3, and Aurora B staining in cardiomyocytes and non-cardiomyocytes from MI rats on day 5, showing enhanced cardiomyocyte proliferation in hearts treated with the R+/miR hydrogel. Reproduced with permission from Wang *et al.*, Biomaterials **312**, 122732 (2025). Copyright 2025 Elsevier.^85^ (b) Schematic illustration of the synthesis of the ROS-responsive CS-B-NO hydrogel and its therapeutic application in myocardial I/R injury. (c) *In vitro* degradation of the CS-B-NO hydrogel with or without H_2_O_2_ (100 *μ*M). The residual weight of the dried hydrogel was recorded at various time points to evaluate degradation behavior. (d) *In vitro* cumulative NO release from CS-B-NO hydrogel (5 mg) in 5 ml PBS (pH 7.4) containing various concentrations of H_2_O_2_. Reproduced with permission from Hao *et al.*, Adv. Sci. **9**, 2105408 (2022). Licensed under a Creative Commons Attribution (CC BY) license/Copyright 2022 Wiley.^86^ (e) ^1^H NMR spectra of 4-arm-PEG-SG and PAMB-G-TK. (f) Release of S1P/SS-31/Lipo from hydrogels at PBS, 0.1, and 0.5 mM H_2_O_2_ concentrations. (g) TEM images of the infarcted mitochondrial structure. Reproduced with permission from Zheng *et al.*, Adv. Healthcare Mater. **11**, 2200990 (2022). Copyright 2022 Wiley.^91^

To achieve targeted drug release within the oxidative stress-enriched microenvironment of MI, some ROS-responsive hydrogels rely on the oxidative cleavage of specific chemical bonds. This section focuses on three commonly used ROS-labile linkages: diselenide, boronate ester, and thioketal. Each type exhibits distinct degradation kinetics, and under elevated ROS levels, these bonds undergo oxidative scission, triggering controlled therapeutic release while also contributing to ROS scavenging and antioxidative effects^81–83^ (Table I). The diselenide (Se–Se) bond is a highly ROS-responsive linkage in smart hydrogel systems. ROS such as H_2_O_2_ oxidizes the Se–Se bond, generating hydrophilic selenic acid that converts the hydrophobic diselenide into water-soluble selenium oxide, leading to rapid network breakdown and drug release^55^ [Fig. 2(D)]. Because of its low bond energy (∼172 kJ mol^−1^), the Se–Se bond reacts sensitively to pathological oxidative stress. The oxidation product, selenic acid, can also catalyze ROS scavenging, providing a self-regulating antioxidant effect.^84^ In contrast, proton-induced degradation serves only as a secondary pathway. The pronounced ROS responsiveness of the diselenide bond enables targeted degradation within infarcted myocardium, making it a promising platform for localized drug delivery and oxidative stress therapy. Wang *et al.* developed an injectable ROS-scavenging hydrogel composed of cyclodextrin (CD)-terminated diselenide hyperbranched polymer (HSe-CD), CD-modified hyaluronic acid (HA-CD), adamantane-modified HA (HA-Ad), and miR-19a/b-cholesterol^85^ (Fig. 4). Due to the high ROS sensitivity of diselenide bonds, the hydrogel injected into the infarcted myocardium gradually degraded, enabling the specific and sustained release of miR-19a/b. This system effectively attenuated inflammation, reduced apoptosis and cardiac fibrosis, and promoted functional recovery in post-MI rat models. At day 5 post-injection in MI rats, Ki67, pH3, and Aurora B staining showed that the R^+^/miR hydrogel markedly increased cardiomyocyte proliferation, confirming the synergistic effect of ROS-scavenging hydrogel and miR-19a/b [Fig. 5(a)]. The synergistic effect between this intelligent responsive hydrogel and the loaded drug or mRNA holds significant innovative potential for improving cardiac function after MI.

As discussed in Sec. II A 2 a, boronate ester bonds are responsive to changes in both pH and ROS. In contrast to reversible pH-dependent hydrolysis, oxidation by ROS is an irreversible process because boric acid, formed as the oxidation product, cannot react with diols to regenerate boronate esters under physiological conditions [Fig. 2(B)]. This mechanism enables continuous drug release once oxidative stress occurs. Moreover, the oxidative degradation pathway itself helps to scavenge excessive ROS in the infarcted myocardium, offering dual therapeutic effects through regulated drug delivery and inherent antioxidant activity. The combined features of pH and ROS responsiveness, irreversible oxidation behavior, and natural ROS-neutralizing ability set boronate ester bonds apart from other ROS-responsive linkages and make them promising candidates for adaptive drug release in cardiac repair. Hao *et al.* developed a hydrogel named CS-B-NO, which possesses dual functions of ROS responsiveness and nitric oxide (NO) release^86^ [Fig. 5(b)]. In this system, boronate ester groups act as protective moieties and react with azide-modified diols through ozonide coupling to form a small-molecule NO donor (B-NO). The resulting B-NO moieties are then grafted onto the side chains of natural chitosan (CS) via a click reaction, yielding the CS-B-NO hydrogel. *In vitro* degradation experiments demonstrated that the CS-B-NO hydrogel remained stable in phosphate-buffered saline (PBS), whereas the presence of H_2_O_2_ accelerated its decomposition, suggesting that oxidative stress facilitates hydrogel degradation [Fig. 5(c)]. With increasing H_2_O_2_ levels, the hydrogel exhibited gradual and sustained NO release, which can be ascribed to the strong ROS sensitivity of the boronate ester bonds [Fig. 5(d)]. This ROS-responsive design enables the hydrogel to both scavenge excessive ROS and release NO in a controlled manner, providing combined antioxidative and therapeutic effects, thereby mitigating myocardial I/R injury. However, the ischemic duration used in this study was limited to 30 min. Future studies could extend the ischemia period to better mimic clinical myocardial I/R conditions and evaluate the therapeutic efficacy of the CS-B-NO hydrogel under more severe ischemic injury. It is also important to determine the optimal therapeutic window for achieving the best treatment outcomes. In another study, Shen *et al.* constructed a melatonin (MLT)-loaded hydrogel by cross-linking polyvinyl alcohol (PVA) with a boronate-containing crosslinker-N^1^-(4-boronobenzyl)-N^3^-(4-boronophenyl)-N^1^, N^1^, N^3^, N^3^-tetramethylpropane-1,3-diamine (TSPBA) via boronate ester bonds.^87^ ROS-induced cleavage of these bonds in infarcted tissue triggered on-demand MLT release, thereby reducing systemic side effects, improving bioavailability, and achieving precise treatment of myocardial I/R injury.

Analogous to boronate esters, the thioketal (TK) bond is an important ROS-responsive linkage characterized by a central quaternary carbon atom flanked by two thioether groups [R_1_SC(CH_3_)_2_–S–R_2_]. This structure has been widely employed in the design of smart hydrogels for myocardial repair^88^ [Fig. 2(C)]. When exposed to ROS, thioketal groups undergo cleavage, generating two thiol compounds: acetone and oxygen. Such ROS-triggered degradation enables controllable drug release and simultaneously scavenges pathological ROS, thereby alleviating oxidative stress. In addition, thioketal synthesis is straightforward, and the resulting materials show excellent biocompatibility.^89^ By tuning the hydrophilicity or hydrophobicity of the thiolated components, the swelling and degradation behavior of the hydrogel can be precisely regulated, making thioketal chemistry a versatile tool for constructing multifunctional delivery systems in cardiac tissue repair.^90^ Zheng *et al.* developed a liposome-composite hydrogel——poly-3-amino-4-methoxybenzoic acid withTK-NH2-modified gelatin (PAMB-G-TK) crosslinked via TK bonds for the co-delivery of mitochondria-targeted SS-31 peptides and sphingosine-1-phosphate (S1P).^91^ The hydrogel precursor solution containing 3% (w/v) PAMB-G-TK and 1.5% (w/v) four-arm-PEG-succinimidyl glutarate ester (PEG-SG) exhibited a gelation time of 30–50 s, making it particularly suitable for intramyocardial injection. The (^1^H NMR) spectrum verified the successful synthesis of PAMB-G-TK [Fig. 5(e)]. *In vitro* release analysis showed that S1P/SS-31/Lipo reached about 95% release under 0.5 mM H_2_O_2_ within 14 days, much higher than at 0.1 mM H_2_O_2_, confirming that liposome release from the hydrogel depends on ROS levels [Fig. 5(f)]. Furthermore, on day 3 after myocardial infarction, TEM analysis showed that treatment with the S1P/SS-31/Lipo-loaded hydrogel markedly improved mitochondrial morphology and integrity, thereby mitigating cardiomyocyte injury [Fig. 5(g)]. In a rat MI model, this hydrogel exhibited pronounced therapeutic efficacy by scavenging excessive ROS, restoring mitochondrial function, and promoting angiogenesis, ultimately enhancing myocardial repair and functional recovery.

In summary, the strategic incorporation of chemical bonds with ROS-labile properties enables the design of hydrogels that undergo site-specific degradation and controlled drug release in oxidative stress environments. These systems provide a promising therapeutic platform for the targeted treatment of MI, combining controlled delivery with intrinsic ROS-scavenging capabilities to achieve synergistic cardioprotection. Despite encouraging outcomes, several challenges limit clinical translation. The balance between ROS elimination and physiological redox signaling remains unclear, and excessive antioxidant activity may hinder macrophage polarization, fibroblast activation, and endothelial migration essential for repair. The proposed synergy between ROS-scavenging hydrogels and therapeutic agents lacks mechanistic support. Moreover, irreversible boronate ester oxidation, though enabling continuous release, fails to adjust to fluctuating oxidative stress after infarction, potentially causing early drug exhaustion and insufficient support during later healing. For true clinical translation to be realized, these challenges need to be critically evaluated and overcome.

##### b. ROS-responsive materials.

Building upon the mechanisms of ROS-labile chemical bonds, another design strategy focuses on the intrinsic physicochemical responsiveness of materials themselves. Polymers possessing both hydrophilic and hydrophobic properties are commonly employed in the design of ROS-responsive hydrogels.^92^ In the absence of ROS, the hydrophobic segments interact to maintain the hydrogel in an aggregated state with low solubility. However, upon exposure to ROS, these hydrophobic domains undergo oxidative reactions that convert them into hydrophilic moieties.^93^ This transformation disrupts the original hydrophobic interactions, thereby increasing the overall hydrophilicity and solubility of the hydrogel.^94^ Consequently, this structural change triggers the release of encapsulated drugs or cytokines. Based on this principle, Luo *et al.* developed a hydrogel composed of a methoxy polyethylene glycol-poly(L-methionine-co-L-alanine) (PMA) copolymer and the immunomodulatory drug FTY720.^95^ At the site of MI, the methionine units within the PMA act as antioxidants, scavenging ROS. Simultaneously, the thioether groups in their side chains are oxidized by ROS, inducing a transition of PMA from hydrophobic to hydrophilic. This gel-to-sol transformation enables controlled, ROS-dependent release of the encapsulated FTY720. Such a release mechanism ensures targeted drug delivery to the site of myocardial reperfusion injury, thereby enhancing drug utilization efficiency. It effectively inhibits cardiomyocyte apoptosis, modulates macrophage polarization, and attenuates inflammation while promoting angiogenesis. Similarly, Wang *et al.* designed an ROS-responsive, crosslinked polyvinyl alcohol-N^1^-(4-boronobenzyl)-N^3^-(4-boronophenyl)-N^1^,N^1^,N^3^,N^3^-tetramethylpropane-1,3-diamine (PVA-TSPBA) hydrogel capable of encapsulating an IL-1β-targeting nanobody.^96^ In the ROS-rich microenvironment of MI, oxidation of the TSPBA moieties led to hydrogel degradation and facilitated the precise, controlled release of the nanobody. The hydrophilic–hydrophobic transition mechanism of ROS-responsive hydrogels offers a versatile strategy for the targeted delivery of drugs and cytokines. By exploiting the elevated ROS concentrations in pathological tissues and the inherent ROS sensitivity of specific polymers, these systems achieve site-specific drug release, improved bioavailability, and enhanced therapeutic efficacy.

Amphiphilic polymer-based ROS-responsive hydrogels achieve effective drug delivery in MI regions through hydrophobic–hydrophilic transitions, leading to notable therapeutic benefits. However, this strategy, which relies on global hydrophobicity changes, may cause premature drug release under mild oxidative stress or incomplete release in severely necrotic areas with uneven ROS distribution. Future studies should quantify the transition kinetics under physiologically relevant ROS gradients and integrate spatial ROS mapping to guide polymer design, enabling delivery dynamics that better match cardiac pathological progression.

##### c. Integration of exogenous ROS scavengers.

Although the cleavage of ROS-sensitive chemical bonds enables targeted drug release and contributes to ROS neutralization, this intrinsic mechanism alone often provides limited antioxidant efficacy. To overcome this limitation, the incorporation of exogenous ROS scavengers into the hydrogel matrix has emerged as an effective strategy.^97,98^ These exogenous agents enhance the overall ROS-clearing capacity of the hydrogel, exert potent antioxidant activity, and synergize with the responsive polymeric network to more effectively modulate the pathological microenvironment.

Metallo-nanozymes, a class of nanomaterials with enzyme-like catalytic activity, typically possess a metal or metal oxide core, such as iron, copper, or manganese nanoparticles.^99^ By mimicking the catalytic functions of natural antioxidant enzymes, these nanozymes exhibit broad application potential in biomedicine.^100^ Hydrogels serve as ideal carriers for the localized delivery and controlled release of metallo-nanozymes, thereby prolonging their retention at infarcted sites and enhancing their antioxidant and tissue-reparative functions.^101^ Metal–organic frameworks (MOFs), such as zeolitic imidazolate framework-8 (ZIF-8), are porous crystalline structures self-assembled from metal ions and organic ligands.^102,103^ MOFs have attracted increasing attention in nanozyme fabrication due to their intrinsic biocompatibility, efficient catalytic activity, and the beneficial role of Zn^2+^ as a catalytic cofactor. Zhong *et al.* designed and synthesized a ZIF-8 nanozyme, which was further incorporated into sodium alginate to prepare a composite hydrogel [ALG-(ZIF-8)].^104^ The resulting hydrogel exhibited both superoxide dismutase (SOD)-like and catalase (CAT)-like enzymatic activities, enabling the efficient elimination of excessive ROS in the MI region. Moreover, ALG-(ZIF-8) effectively suppressed inflammatory responses, promoted angiogenesis, and modulated collagen remodeling, ultimately leading to improved cardiac function. Building upon this, Xu *et al.* proposed a biomimetic strategy by integrating polyphenols with metallo-nanozymes to construct tannic acid-coated Mn-Co_3_O_4_ (MCT) nanoparticles, which were embedded within an injectable collagen hydrogel.^105^

Beyond their potent ROS-scavenging capability, the MCT nanoparticles targeted mitochondria to suppress excessive ROS generation. This dual-functional system significantly reduced cellular apoptosis and inflammation while enhancing cardiac performance, offering new insights into the synergistic integration of nanozymes and natural biomaterials for myocardial repair.

Glutathione (GSH), an endogenous antioxidant composed of glutamate, cysteine, and glycine, plays a crucial role in ROS scavenging within biological systems.^106^ Its incorporation into hydrogel matrices can effectively suppress ROS generation and alleviate oxidative stress. However, the thiol group (–SH) in GSH exhibits high chemical reactivity and is readily affected by environmental factors such as light, temperature, and oxygen, leading to a reduction in antioxidant activity.^107^ To overcome this limitation, chemical modification strategies such as disulfide bond formation and the introduction of protective groups have been employed to enhance GSH stability.^108^ Li *et al.* further grafted GSH onto chitosan (CS) chains via amide bonds and combined it with a zero-length bioactive crosslinker to synthesize CSCleGSH copolymers.^109^ These copolymers were subsequently mixed with glycerophosphate and hydroxyethyl cellulose to fabricate an injectable CSCleGSH hydrogel. This design markedly improved the thermal stability of GSH, allowing it to maintain antioxidant activity within the hydrogel network. As a result, the system effectively inhibited oxidative stress-induced cardiomyocyte damage, demonstrating substantial potential for myocardial repair applications.

ROS-responsive hydrogels can further enhance antioxidant capacity by incorporating exogenous scavengers beyond conventional bond-cleavage mechanisms. However, the potential metal ion toxicity arising from sustained Zn^2+^/Mn^2+^ release and the impact of GSH modification on redox-cycling efficiency remain insufficiently studied. Future research should quantify the optimal scavenging threshold, evaluate the long-term toxicity associated with metal accumulation, and develop feedback-regulated systems that dynamically modulate antioxidant activity according to real-time ROS levels rather than continuous depletion.

### C. MMP-responsive hydrogels

#### 1. MMP dynamics in the MI microenvironment

MMPs are a family of zinc-dependent endopeptidases secreted by various cell types, including fibroblasts, vascular smooth muscle cells (VSMCs), and leukocytes.^110^ They play a crucial role in the degradation and remodeling of the ECM.^111^ Under pathological conditions such as MI, fibrotic diseases, osteoarthritis, and cancer, excessive MMP expression becomes a major contributor to tissue damage and disease progression.^112,113^ This dysregulation disturbs ECM homeostasis and accelerates pathological remodeling.

During MI, ischemia and hypoxia induce extensive apoptosis and necrosis of cardiomyocytes, subsequently initiating an inflammatory response. Inflammatory cells, including macrophages and neutrophils, infiltrate the infarcted region and release pro-inflammatory cytokines such as Tumor Necrosis Factor (TNF-α) and Interleukin-1 (IL-1), which stimulate the expression and activity of MMPs.^114,115^ As primary ECM-degrading enzymes, MMPs exhibit markedly elevated expression during MI, leading to excessive ECM degradation.^116^ Elevated MMP levels further amplify inflammatory cytokine production, forming a positive feedback loop that perpetuates MMP activation.^117,118^ However, sustained inflammatory stimulation also induces increased expression of tissue inhibitors of metalloproteinases (TIMPs), ultimately reducing the MMP/TIMP ratio and promoting maladaptive cardiac remodeling.^114^ Excessive ventricular remodeling causes ventricular wall thinning and dilation, severely compromising cardiac structure and function, leading to systolic and diastolic dysfunction.^119^ Moreover, MMPs regulate scar formation and myocardial fibrosis; dysregulated scarring impairs cardiac electrophysiological stability and mechanical integrity, thereby increasing the risk of arrhythmias and cardiac rupture.^120^ Consequently, targeting MMP activity represents a promising therapeutic strategy for MI, holding substantial clinical potential.

#### 2. Design principles of MMP-responsive hydrogels

**FIG. 6.**
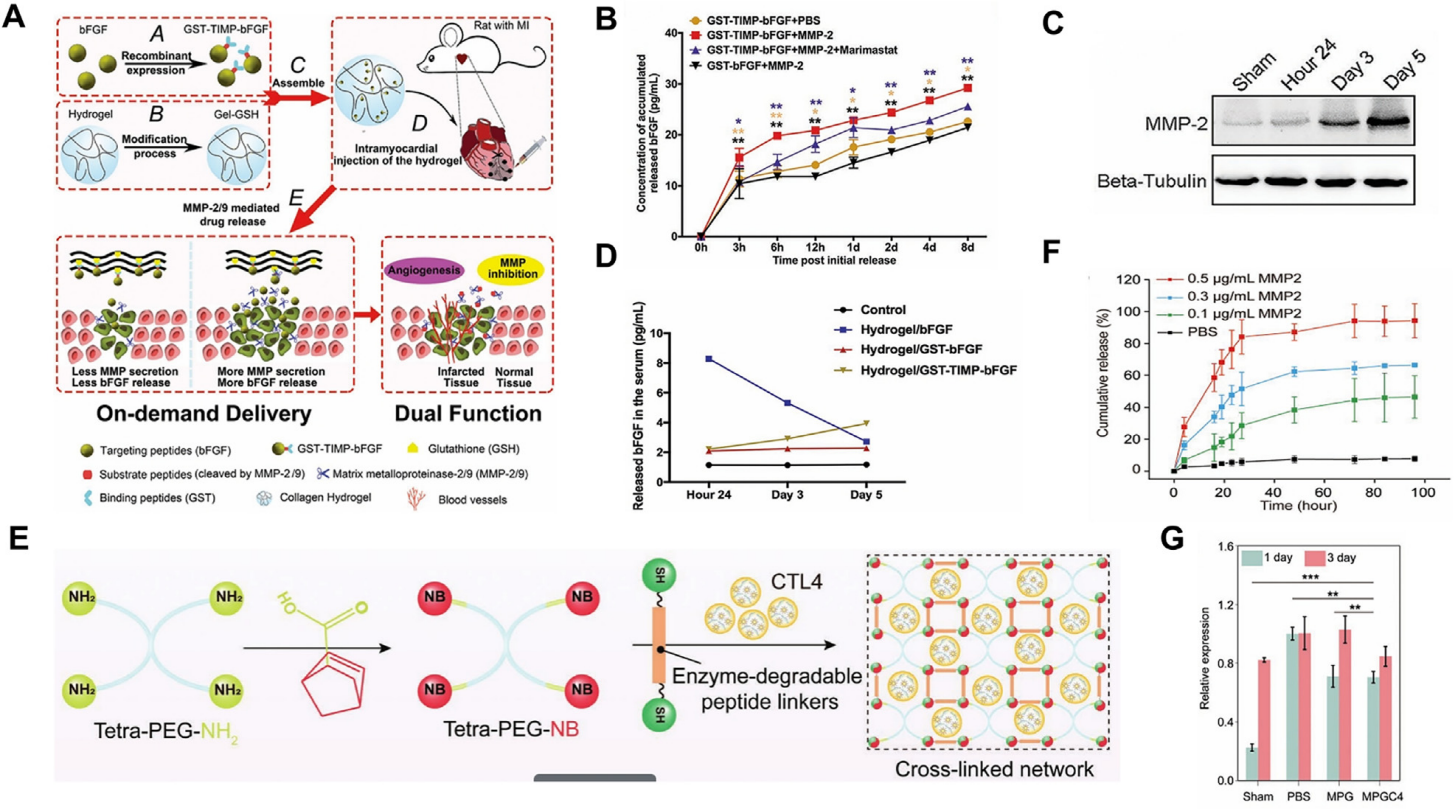
Application of MMP-responsive hydrogels in the treatment of MI. (a) Schematic illustration of the preparation and MMP-responsive drug release mechanism of the Gel/GST-TIMP-bFGF hydrogel in a rat MI model. The fusion protein GST-TIMP-bFGF was prepared via recombinant expression and mixed with Gel-GSH (constructed by chemically cross-linking GSH), utilizing the specific GST–GSH interaction for coupling. Upon injection into the infarcted site, the hydrogel responds to upregulated MMP-2/9, leading to matrix degradation and the release of GST-TIMP-bFGF. The released protein subsequently inhibits MMP activity and promotes angiogenesis to treat myocardial infarction. (b) *In vitro* cumulative release kinetics of bFGF from the hydrogel system mediated by MMP-2 enzymatic degradation at various time points. (c) Western blot analysis showing the progressive upregulation of MMP-2 expression at different time points post-MI. (d) Cumulative bFGF release from the MMP-responsive hydrogel *in vivo* at various time points. Reproduced with permission from Fan *et al.*, Adv. Mater. **31**, 1902900 (2019). Copyright 2019 Wiley.^124^ (e) The synthesized four-armed polyethylene glycol-norbornene (tetra-PEG-NB) was dissolved with MMP-sensitive peptides in CTL4 solution to form the MPGC4 hydrogel system with a crosslinked network structure. (f) Cumulative release curves of CTL4 from MPGC4 hydrogel in MMP-2 solutions at different concentrations. (g) Quantitative PCR analysis of MMP-2 mRNA expression levels at days 1 and 3 following MI. Reproduced with permission from Chen *et al.*, Adv. Mater. **35**, 2209041 (2023). Copyright 2023 Wiley.^125^

TIMPs, a critical class of regulatory proteins, primarily modulate MMP activity to maintain ECM homeostasis.^121^ They have been extensively investigated for treating adverse tissue remodeling driven by excessive MMP activity.^122^ By incorporating MMP-specific peptide sequences into hydrogels and loading TIMP-3 or therapeutic agents, site-specific degradation can be achieved exclusively in regions with elevated MMP expression.^123^ This strategy enables localized MMP inhibition, thereby modulating the pathological microenvironment and enabling targeted therapy. Building on this principle, Fan *et al.* engineered a dual-functional intelligent responsive hydrogel for on-demand growth factor release and MMP inhibition in MI zones^124^ [Fig. 6(a)]. The hydrogel, composed of collagen and GSH, was loaded with the fusion protein GST-TIMP-bFGF. In infarcted myocardium where the MMP-2/9 expression is upregulated, the TIMP peptide undergoes specific cleavage, releasing bFGF to promote angiogenesis and significantly improve post-infarction cardiac function. *In vitro* release studies demonstrated that bFGF release from the GST-TIMP-bFGF group was significantly higher than that observed in the absence of MMP-2 or in the presence of the MMP-2 inhibitor marimastat, confirming the hydrogel system's specific MMP-2-responsive release behavior [Fig. 6(b)]. Furthermore, bFGF release from the GST-TIMP-bFGF hydrogel gradually increased from day 3 to day 5, aligning with the upregulation pattern of MMP-2 following MI. This correlation further demonstrates that the hydrogel system dynamically responds to MMP-2 levels in the microenvironment to achieve sustained bFGF release [Figs. 6(c) and 6(d)]. Although collagen served as the biomaterial scaffold in this study, future investigations should consider integrating materials with improved biocompatibility, controllable degradation profiles, and optimized mechanical properties to enhance therapeutic efficacy. Furthermore, engineering drug release systems responsive to specific MMP subtypes could provide greater spatiotemporal control over therapeutic agent delivery. Similarly, Chen *et al.* developed an intelligent hydrogel system named MPGC4, which is composed of CTL4 consisting of carbon dots (CDots) and interleukin-4 plasmid DNA (IL4-pDNA), and an MMP-2/9-responsive polyethylene glycol (PEG) hydrogel (MPG)^125^ [Fig. 6(e)]. CTL4, serving as a composite gene nanocarrier, was encapsulated within the MPG network through a photopolymerization reaction. The crosslinked structure of MPG contains MMP-sensitive peptide chains, which enable the targeted release of CTL4 in the MI region with elevated MMP-2/9 expression. As the solid content of the hydrogel system increased, the gelation time decreased. At an 8% concentration of MPG, the gelation time was approximately 63 s, indicating a rapid sol–gel transition. *In vitro* assays demonstrated concentration-dependent acceleration of CTL4 release in response to increasing MMP-2 levels, confirming the enzyme-specific degradation mechanism of MPGC4 [Fig. 6(f)]. The released CTL4 from the degradable hydrogel system effectively inhibits MMP-2/9 expression [Fig. 6(g)], facilitates M2 macrophage polarization, reduces inflammatory responses, enhances therapeutic angiogenesis, and ultimately restores cardiac function.

**FIG. 7.**
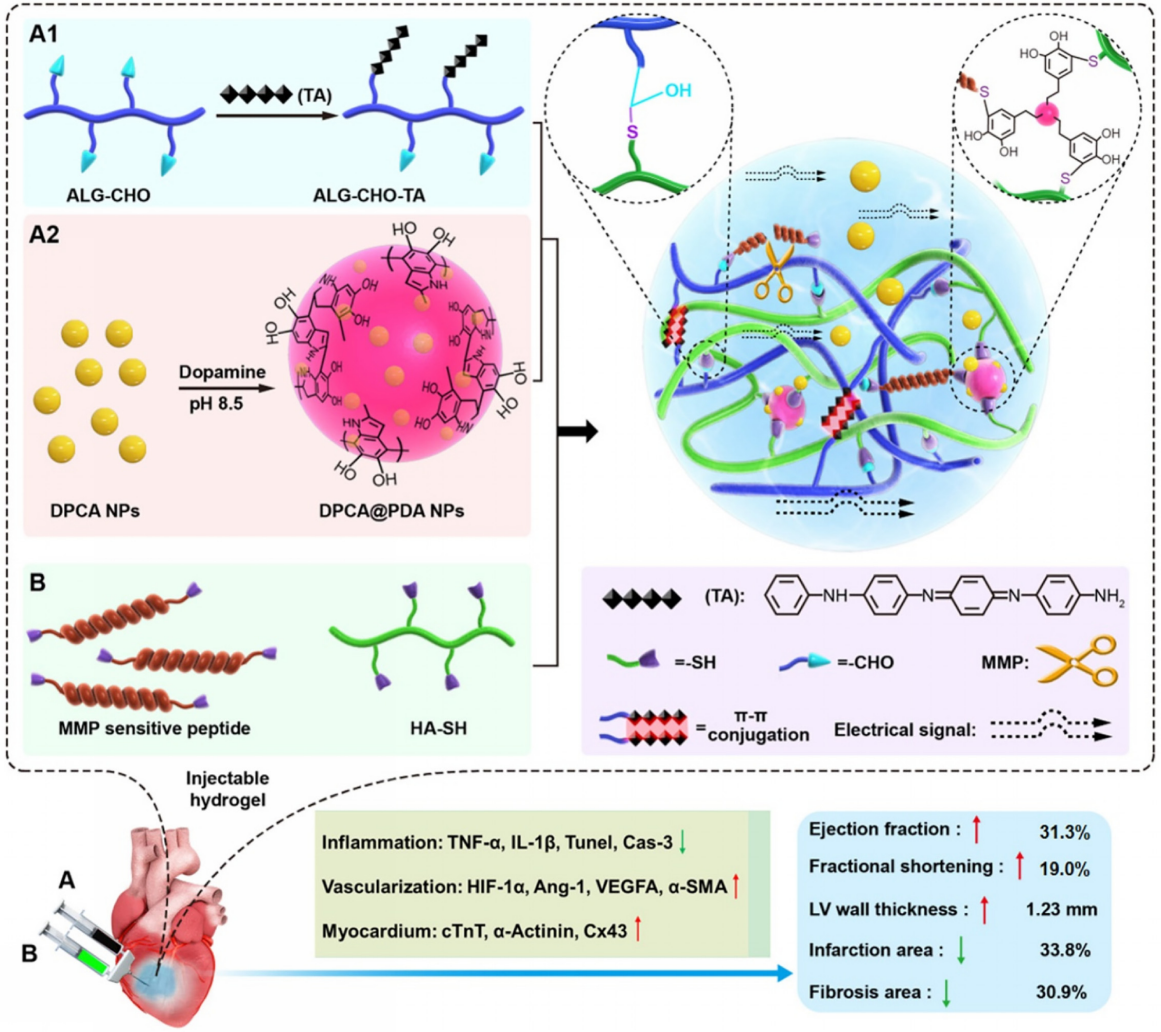
Schematic diagram showing the fabrication of the ALG-CHO-TA/DPCA@PDA/MMP-SP/HA-SH hydrogel system and its therapeutic treatment following injection into the infarcted rat heart. The hydrogel was fabricated from functionalized HA and ALG, with TA incorporated into ALG-CHO for electrical conductivity and PDA-coated DPCA nanoparticles for high drug loading and sustained release. The multifunctional network was formed via cross-linking of ALG-CHO and DPCA@PDA with HA-SH and thiolated MMP-SP. When injected into the infarcted myocardium, the system provides intelligent, sustained DPCA release that stabilizes HIF-1α, thereby conferring multiple therapeutic benefits including inflammation suppression, angiogenic promotion, fibrosis reduction, and cardiac functional improvement. Reproduced with permission from Wei *et al.*, Theranostics **12**(1), 127–142 (2022). Licensed under a Creative Commons Attribution (CC BY) license/Copyright 2022 Ivysping International Publisher.^126^

Additionally, Wei *et al.* designed an injectable hydrogel integrating hyaluronic acid (HA), oxidized alginate (ALG-CHO), tetraaniline (TA), and DPCA nanoparticles.^126^ Here, TA imparts electrical conductivity, DPCA stabilizes HIF-1α, while ALG-CHO and HA provide structural support. The hydrogel was functionalized with MMP-sensitive peptides (MMP-SP) and polydopamine (PDA), enabling MMP-responsive degradation and sustained DPCA release. Following intramyocardial administration in infarcted rats, this system enhanced cardiac conductivity, significantly improved cardiac function, reduced fibrosis, and promoted angiogenesis (Fig. 7). To advance clinical translation, future studies must address several key challenges: optimizing the physicochemical properties and delivery kinetics of the hydrogel system, and comprehensively investigating the molecular mechanisms governing DPCA-mediated HIF-1α stabilization and its associated signaling cascades in the context of MI therapy.

An alternative approach to controlled TIMP-3 delivery exploits the intrinsic biochemical properties of the protein itself. The C-terminal domain of TIMP-3 contains an abundance of lysine and arginine residues, which confer a net positive charge. These positively charged amino acids facilitate electrostatic interactions with the negatively charged sulfate groups on glycosaminoglycans, enabling the formation of stable molecular complexes.^127^ Leveraging this property, Burdick and colleagues developed an innovative strategy by engineering dextran sulfate (DS) as an anionic polymer and chemically modifying hyaluronic acid (HA) to introduce aldehyde (ALD) and hydrazide (HYD) functional groups.^128^ An injectable hydrogel network was constructed by conjugating hydrazide-terminated MMP-degradable peptides with negatively charged hyaluronic acid (HYD-HA), followed by cross-linking with aldehyde-functionalized hyaluronic acid (ALD-HA) through a Schiff base reaction. In this system, recombinant TIMP-3 (rTIMP-3), bearing a net positive charge, was stably incorporated into the hydrogel matrix through electrostatic interactions with the anionic polysaccharides. Upon intramyocardial injection, the elevated MMP activity in the infarcted myocardium specifically cleaved the MMP-sensitive peptide linkers, thereby disrupting the hydrogel's crosslinked structure and enabling controlled release of rTIMP-3. This targeted release strategy effectively suppressed MMP activity within the infarct zone and led to significant improvements in post-infarction left ventricular function.

Beyond protein and drug release applications, MMP-responsive hydrogels have also emerged as promising platforms for stem cell-based therapies in MI treatment.^129^ Human induced pluripotent stem cells (iPSCs), generated by reprogramming somatic cells to an embryonic stem cell-like pluripotent state, are widely utilized in cell therapy and tissue engineering. Effective cardiac repair using iPSCs requires precise differentiation into cardiomyocytes; however, current technologies struggle to achieve complete directed differentiation. Upon transplantation into infarcted myocardium, residual undifferentiated iPSCs may undergo aberrant proliferation and form tumors.^130^ Furthermore, challenges persist in achieving electrophysiological and structural integration between iPSC-derived cardiomyocytes (hiPS-CMs) and host cardiomyocytes, potentially leading to arrhythmic complications.^131^ Thus, critical modifications are essential to enhance the quality and safety of hiPS-CMs prior to clinical application for MI. To overcome these limitations, Li *et al.* developed an AuNP/HA composite hydrogel matrix loaded with hiPS-CMs.^132^ The hydrogel is based on a methacrylate-modified HA backbone and crosslinked via an MMP-2-cleavable peptide, enabling *in situ* degradation in response to the elevated MMP-2 activity present in ischemic myocardium. RGD peptides were incorporated to enhance biocompatibility. The incorporation of AuNPs is intended to modulate the stiffness and topological structure of the nanomatrix, thereby promoting gap junction formation in hiPS-CMs and regulating calcium handling through the αnβ1 integrin-mediated ILK-1/p-AKT/GATA4 signaling pathway. The results demonstrated that this hydrogel system improved cardiomyocyte electrophysiological and contractile function, enhanced cardiac performance in MI mice, reduced fibrosis, and promoted angiogenesis, thereby offering a novel biomaterial strategy for cardiac repair using hiPS‐CMs. However, this study only examined a single hiPS-CM dose, making it difficult to fully assess arrhythmia risk and long-term graft survival due to the short observation period. Future studies will optimize the AuNP-HA hydrogel, evaluate the safety of gradient hiPS-CM transplantation, and establish precise cell-tracking methods for real-time monitoring of cell fate.

Collectively, these studies underscore the versatility and therapeutic potential of MMP-responsive hydrogels in myocardial repair. Whether through the localized delivery of proteins, genes, small molecules, or stem cells, these intelligent biomaterials offer spatiotemporal control over therapeutic release and precise microenvironment modulation. However, it is worth noting that complete inhibition of MMP activity may impair the physiological tissue remodeling and angiogenesis essential for cardiac repair. Future studies should define the therapeutic window for MMP inhibition and integrate advanced cell-tracking technologies, such as bioluminescence imaging and MRI-detectable cell labeling, together with computational models based on patient-specific MMP profiles to predict release kinetics, ultimately enabling personalized biomaterial-based cardiac therapy.

### D. Thermoresponsive hydrogels

Human body temperature exhibits regional heterogeneity, with pathological tissues often displaying distinct thermal profiles compared to adjacent healthy regions.^133^ This physiological characteristic offers a unique advantage for the application of thermosensitive hydrogels in targeted drug delivery and controlled therapeutic release.^134^ Temperature-sensitive polymers, as thermoresponsive macromolecular materials, can undergo a rapid and reversible sol–gel phase transition near their lower critical solution temperature (LCST), which is close to human body temperature, and this process can be precisely regulated by temperature.^135,136^ These polymers are typically composed of both hydrophilic and hydrophobic functional groups. Such polymers typically contain hydrophobic groups (e.g., methyl, ethyl, and propyl) and hydrophilic groups (e.g., hydroxyl, carboxyl, and amide).^137^ The interactions among these groups, particularly hydrophobic interactions and hydrogen bond formation, are highly sensitive to temperature variations.^138^ When the ambient temperature falls below the LCST, enhanced hydrophobic interactions and increased hydrogen bonding promote hydrogel network contraction, leading to a reduction in volume. In contrast, temperatures above the LCST weaken hydrophobic interactions, resulting in network relaxation and volume expansion.^139^ This thermally responsive behavior enables rapid *in situ* gelation at physiological temperatures, making thermosensitive hydrogels highly adaptable to the dynamic cardiac microenvironment. This characteristic enables the hydrogel to rapidly gelate at body temperature, thereby adapting to the physiological environment of myocardial tissue.

**FIG. 8.**
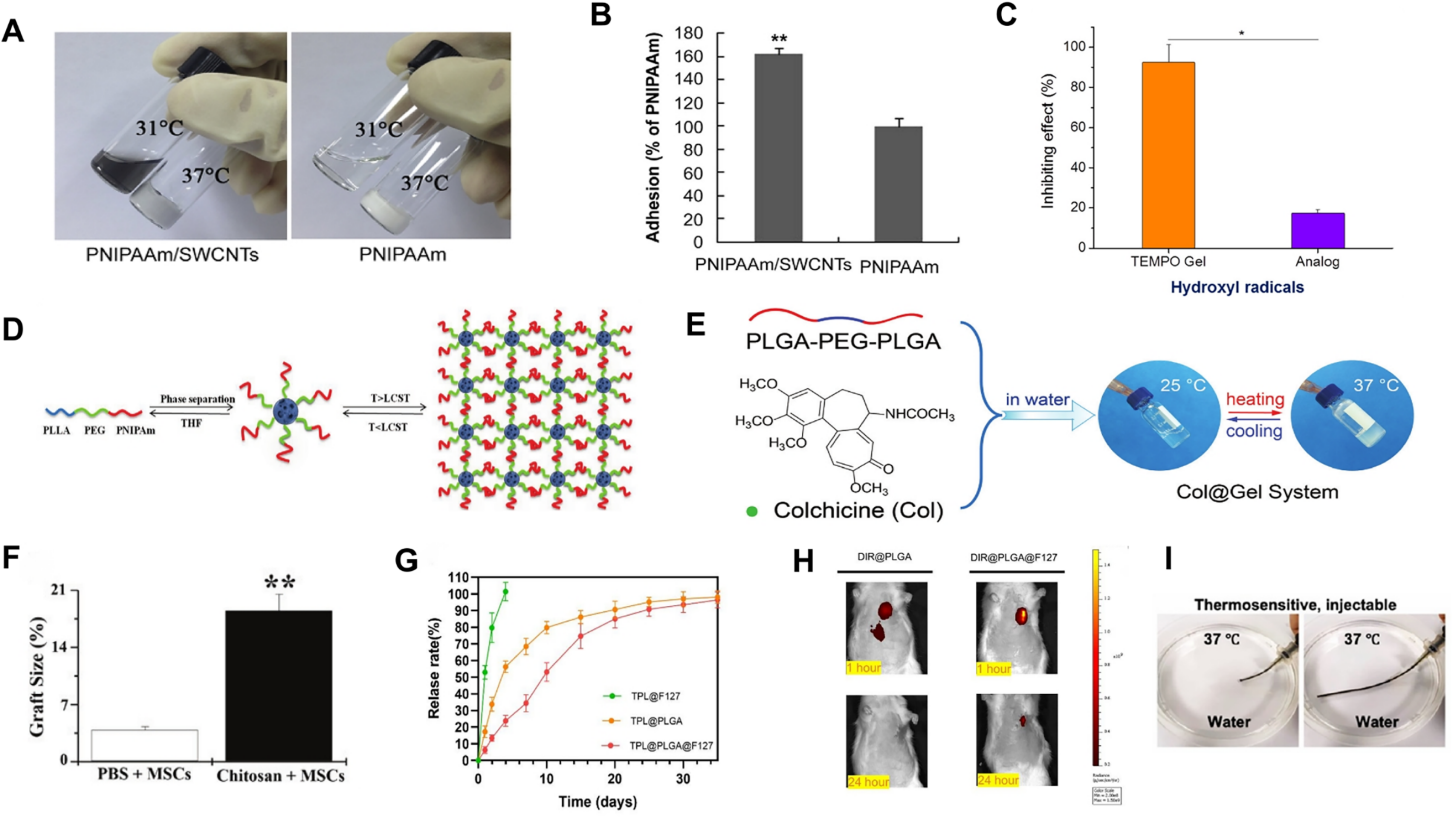
Application of thermoresponsive hydrogels in the treatment of MI. (a) Morphological states of PNIPAAm/SWCNTs and PNIPAAm hydrogels at 31 and 37 °C. Both hydrogels exhibit temperature-responsive behavior, remaining in a sol (liquid) state at 31 °C and transforming into a gel state at 37 °C. (b) Quantitative analysis of BASC adhesion on PNIPAAm/SWCNTs and PNIPAAm hydrogels under ROS conditions. Reproduced with permission from Li *et al.*, Biomaterials **35**, 5679–5688 (2014). Copyright 2014 Elsevier.^140^ (c) Quantitative analysis of the hydroxyl radical scavenging ability of the hydrogels determined by the pyrogallol assay. Reproduced with permission from Zhu *et al.*, Biomaterials **177**, 98–112 (2018). Copyright 2018 Elsevier.^141^ (d) The PLLA–PEG–PNIPAAm triblock copolymer self-assembled into nanofibrous microspheres (NF-GMS), which subsequently formed hydrogels through thermo-induced physical cross-linking. Reproduced with permission from Zhao *et al.*, Adv. Funct. Mater. 30, 2000776 (2020). Copyright 2020 Wiley.^142^ (e) Schematic illustration of the fabrication and thermoresponsive behavior of the Col@Gel system. At 25 °C, the PLGA–PEG–PLGA-based sol remains flowable, whereas at physiological temperature (37 °C), it undergoes spontaneous and reversible sol-to-gel transition, forming a semi-solid hydrogel suitable for *in situ* application. Reproduced with permission from Chen *et al.*, J. Mater. Chem. B **8**(5), 980–992 (2020). Copyright 2019 Royal Society of Chemistry.^143^ (f) Quantitative analysis of mesenchymal stem cells (MSCs) in the infarcted myocardium 24 h after hydrogel injection. Reproduced with permission from Xu *et al.*, Exp. Ther. Med. **13**, 588–594 (2017). Licensed under a Creative Commons Attribution (CC BY) license/Copyright 2017 Spandidos Publications.^144^ (g) *In vitro* release profiles of TPL@F127, TPL@PLGA, and TPL@PLGA@F127 formulations. TPL@PLGA@F127 demonstrated the slowest and most controlled release behavior. (h) *In vivo* fluorescence imaging showing cardiac retention of DIR@PLGA and DIR@PLGA@F127 nanoparticles. The incorporation of F127 hydrogel significantly prolonged the *in vivo* retention of PLGA nanoparticles. Reproduced with permission from Wang *et al.*, J. Nanobiotechnol. **21**, 227 (2023). Licensed under a Creative Commons Attribution (CC BY) license/Copyright 2023 Springer.^32^ (i) Schematic illustration of the *in vitro* thermogelation behavior of the aqueous hydrogel solution. Reproduced with permission from Zhang *et al.*, ACS Nano **18**, 10216–10229 (2024). Copyright 2024 ACS Publications.^145^

Poly(N‐isopropylacrylamide) (PNIPAAm) hydrogels, widely utilized as thermosensitive materials, serve as versatile carriers in therapeutic delivery systems.^146^ Li *et al.* fabricated single-walled carbon nanotube (SWCNT)‐modified PNIPAAm hydrogels, which not only enhanced cellular adhesion and proliferation but also demonstrated therapeutic efficacy in MI.^140^ In their study, brown adipose‐derived stem cells (BASCs) were encapsulated within the hydrogel and injected into the infarcted regions of rat hearts. Notably, the PNIPAAm hydrogel remained in a liquid state below 32 °C, facilitating injection and cell encapsulation, whereas it rapidly transitioned to a gel state at 37 °C, providing structural support for the embedded cells [Fig. 8(a)]. In addition, SWCNT modification provided a more favorable microenvironment for transplanted cell adhesion and survival under ROS conditions, thereby enhancing stem cell-mediated cardiac repair [Fig. 8(b)]. Similarly, Zhu *et al.* synthesized a thermoresponsive injectable hydrogel termed TEMPO Gel, composed of poly (N‐isopropylacrylamide‐co‐vinyl pyrrolidone‐co‐polylactic acid methacrylate‐co‐TEMPO methacrylate) (pNVMT).^141^ This material exhibited exceptional antioxidant properties, efficiently scavenging multiple ROS to mitigate I/R injury [Fig. 8(c)]. Concurrently, it improved post‐infarction left ventricular geometry and promoted angiogenesis. Zhao *et al.* designed biodegradable poly(L‐lactide)‐b‐poly(ethyleneglycol)‐b‐poly(N‐isopropylacrylamide) (PLLA‐PEG‐PNIPAAm) triblock copolymers, which self‐assembled into nanofibrous gelatin microspheres (NF‐GMS)^142^ [Fig. 8(d)]. These NF‐GMS exhibited excellent cell adhesion and thermoresponsiveness, undergoing rapid hydrolysis‐driven hydrophobic transitions at 37 °C to form three‐dimensional hydrogels that provided structural support and protection to cardiomyocytes. Furthermore, co‐transplantation of human embryonic stem cell‐derived cardiomyocytes (hESC‐CMs) with NF‐GMS into infarcted rat hearts significantly improved cell survival, promoted angiogenesis, reduced infarct size, and restored cardiac function.

The triblock copolymer hydrogel composed of poly (lactic-co-glycolic acid)–polyethylene glycol–poly (lactic-co-glycolic acid) (PLGA–PEG–PLGA) has been widely investigated for drug delivery in various diseases, including cancer, diabetes, and osteomyelitis, owing to its excellent thermosensitivity, biocompatibility, and biodegradability.^147,148^ This hydrogel undergoes a sol–gel transition at physiological temperature and exhibits favorable injectability, gel-forming ability, and drug-loading capacity, making it particularly advantageous for sustained-release delivery systems.^149,150^ However, its application in the treatment of MI remains relatively underexplored. Chen *et al.* developed a PLGA–PEG–PLGA-based hydrogel network system (Col@Gel) encapsulating colchicine as a therapeutic agent.^143^ The system remains in a free-flowing sol state at 25 °C, facilitating local myocardial injection, and undergoes a sol–gel transition at 35 °C to form a stable gel at 37 °C, thereby enabling sustained and localized colchicine release [Fig. 8(e)]. Both *in vitro* and *in vivo* studies demonstrated that this hydrogel system effectively inhibited post-MI inflammation, apoptosis, and myocardial fibrosis, improved cardiac function, and enhanced survival rates in animal models while significantly reducing the systemic toxicity of colchicine. These findings highlight the promising potential of PLGA–PEG–PLGA hydrogels in the treatment of MI.

Thermosensitive chitosan hydrogels have emerged as ideal carriers for stem cell transplantation, demonstrating distinct advantages in preclinical studies.^151–153^ Embryonic stem cells (ESCs) hold significant promise for MI therapy due to their pluripotency and capacity to differentiate into cardiomyocytes.^154^ However, the clinical efficacy of ESC transplantation is constrained by poor post-transplantation survival rates and suboptimal differentiation efficiency.^155^ To address these limitations, Lv *et al.* investigated the combinatorial delivery of somatic cell nuclear transfer (SCNT)-derived and fertilization-derived ESCs via thermosensitive chitosan hydrogels into infarcted rat hearts.^156^ Their findings revealed that, compared with ESC transplantation alone, hydrogel-encapsulated ESCs exhibited significantly enhanced retention and increased graft size within the infarcted myocardium. Notably, SCNT-ESC delivery via hydrogels markedly improved cardiac function and significantly increased arterial and venous density in the infarct zone. Similarly, Xu *et al.* utilized thermosensitive chitosan hydrogels to deliver mesenchymal stem cells (MSCs) to the post-infarction myocardium in rats.^144^ This strategy substantially improved MSC retention in the infarcted tissue [Fig. 8(f)], promoted their differentiation into cardiomyocytes, and enhanced pro-angiogenic effects, leading to significant improvements in cardiac function and hemodynamic parameters in MI rats. This approach provides a novel clinical paradigm for MSC-based MI therapy. In a more advanced design, Niu *et al.* developed a series of injectable, photoluminescent, thermosensitive, rapid-gelling, highly flexible, and biodegradable hydrogels.^157^ These materials were synthesized by copolymerizing NIPAAm, 2-hydroxyethyl methacrylate (HEMA), 1-vinyl-2-pyrrolidone (VP), and acrylic acid–oligolactide (AOLA), followed by conjugation with hypericin (HYP) to confer photoluminescent properties. The hydrogels enabled targeted stem cell delivery to infarcted cardiac and skeletal muscle tissues. Both *in vitro* and *in vivo* studies demonstrated excellent biocompatibility, stimulation of MSC proliferation and paracrine activity, and suitability as stem cell carriers. Critically, HYP-mediated photoluminescence allowed noninvasive tracking of hydrogel distribution and degradation via fluorescence imaging. This integrated approach represents a promising strategy for advancing stem cell therapies through real-time monitoring and optimized delivery.

Poloxamer 407 (Pluronic F-127), an FDA-approved triblock copolymer (PEO–PPO–PEO), exhibits thermosensitive gelation, excellent biocompatibility, and drug delivery capabilities.^158^ At low temperatures (25 °C), Pluronic F-127 exists as a free-flowing liquid.^159^ Upon exposure to physiological temperature, it undergoes a sol–gel transition, where loose micelles reorganize into a close-packed crystalline-like structure. At concentrations ≥15–20 wt. %, this forms a continuous hydrogel network, enabling precise injectable delivery to infarcted regions for controlled drug release.^160^ Triptolide (TPL), a bioactive compound isolated from *Tripterygium wilfordii*, possesses anti-inflammatory, antitumor, and immunomodulatory properties.^161^ Studies further demonstrate its ability to inhibit NF-κB activity and expression, significantly attenuating cardiac inflammation and fibrosis.^162^ However, systemic administration of TPL induces notable hepatotoxicity and nephrotoxicity, limiting its clinical utility.^163^ To address this, Wang *et al.* engineered a dual sustained-release system (TPL@PLGA@F127) comprising TPL-loaded PLGA nanoparticles embedded in Pluronic F-127 hydrogel.^32^ This system achieved slow, stable TPL release with enhanced myocardial retention, mitigating off-target toxicity [Figs. 8(g) and 8(h)]. Mechanistically, the system modulated macrophage polarization toward the anti-inflammatory M2 phenotype, thereby suppressing cardiomyocyte apoptosis, ameliorating the post-infarction inflammatory microenvironment, inhibiting myocardial fibrosis, improving cardiac function, and preventing adverse ventricular remodeling. Similarly, Zhang *et al.* developed an injectable, highly flexible, conductive composite hydrogel by encapsulating hydrophobic α-tocopherol (α-TOH) within diacrylate-functionalized Pluronic F-127 micelles and incorporating *in situ*-synthesized PDA with conductive components.^145^ This multicomponent hydrogel maintained its thermosensitive property, showing a rapid sol‐to‐gel transition when exposed to water at 37 °C [Fig. 8(i)]. Moreover, this multifunctional hydrogel effectively improved the post-infarction microenvironment, promoted myocardial repair, and enhanced therapeutic outcomes for MI.

Thermoresponsive hydrogels have emerged as optimal candidates for intramyocardial injection owing to their distinctive physicochemical properties. Current thermosensitive hydrogel therapies, though promising, remain largely in the preclinical validation stage. Existing models tend to oversimplify factors such as tissue temperature uniformity, mechanical behavior, and passive degradation. Future research will emphasize the development of integrated, hierarchically responsive platforms capable of staged and precise drug release. At the same time, efforts will focus on designing mechanically adaptive networks with dynamically tunable stiffness. Guided by artificial intelligence, treatment strategies will be tailored to the unique infarction characteristics of individual patients. Ultimately, the goal is to transform these materials from passive delivery carriers into intelligent, self-adaptive therapeutic systems that work in concert with the body's intrinsic cardiac regeneration processes.

### E. Multi-stimuli-responsive hydrogels

The pathological microenvironment at the infarcted site following MI is characterized by pronounced dynamic complexity and heterogeneity. While single-stimulus responsive hydrogels can achieve targeted drug delivery under specific triggers, their therapeutic efficacy often remains limited due to the multifaceted nature of the pathological milieu. In contrast, multi-stimulus responsive hydrogels exploit diverse environmental cues within the infarcted region to achieve spatiotemporally controlled release and precise localization of therapeutic agents or cells. This strategy enhances the local bioavailability of therapeutics, amplifies treatment efficacy, and holds substantial promise for advancing MI therapy.

Among the most extensively studied systems are thermo/pH dual-responsive hydrogels, which undergo specific structural transformations in the acidic and temperature-sensitive infarcted microenvironment. These systems enable controlled, phase-specific drug release, achieving rapid therapeutic action during the early inflammatory phase and sustained release throughout later fibrotic stages. Furthermore, the incorporation of multiple stimuli-responsiveness confers greater functional stability amid physiological variability. When one trigger is weakened by patient-specific factors, alternative stimuli maintain therapeutic efficacy, ensuring continuity in cardiac repair.

For instance, Jiang *et al.* developed a pH/thermoresponsive injectable hydrogel [P(CS-CA-NIPAM)] for localized oncostatin M (OSM) delivery.^164^ The N-isopropylacrylamide (NIPAM) units enabled *in situ* gelation at body temperature, while acid-triggered swelling in ischemic myocardium facilitated sustained OSM release. Following injection into MI rats, this system significantly improved cardiac function, reduced infarct size, and promoted cardiomyocyte proliferation, angiogenesis, and anti-fibrotic effects. Zhang *et al.* developed a pH/ROS dual-responsive injectable conductive hydrogel by dual cross-linking of xanthan gum (OXP) and gelatin (GD) through reversible imine and borate ester bonds, with the incorporation of conductive composites to enhance electrical conductivity and mechanical strength.^28^ By encapsulating polydopamine rosmarinic acid nanoparticles (PDA-RA NPs) within the hydrogel, an on-demand release of RA was achieved through the synergistic pH and ROS responsiveness. This system enables RA to exert multifunctional therapeutic effects, including anti-inflammatory, anti-apoptotic, and anti-fibrotic activities, at different stages of MI, thereby effectively improving cardiac function and promoting myocardial repair. In another study, Zhang *et al.* designed a dual dynamic covalent hydrogel encapsulating mitochondria-targeted, ROS-scavenging nanoparticles (PLGA-TK-PEG-SS31) and cyclosporine A (CsA).^165^ Constructed from diol/hydrazide-modified hyaluronic acid (HA-Diol-HYD) and aldehyde/FPBA-modified HA (HA-FPBA-ALD) via Schiff base and boronate crosslinks, the system responded to low pH and high ROS levels in MI/R hearts. This dual sensitivity enabled controlled nanoparticle release to inhibit mitochondrial apoptotic pathways, thereby protecting cardiomyocytes. Finally, Ding *et al.* synthesized a dual-responsive hydrogel via thiol-ene click chemistry, coupling pH-responsive allyl-modified chitosan (OAL-CS) with thermoresponsive PNIPAM.^166^ Precise swelling control under pH and temperature stimuli, combined with excellent cytocompatibility, rapid gelation, and minimal inflammation, positions this system as a promising injectable platform for controlled drug delivery and minimally invasive tissue engineering applications.

Together, these studies underscore the transformative potential of multi-stimulus responsive hydrogels as next-generation therapeutic platforms for MI. Based on the aforementioned studies, the design strategies of different intelligent responsive hydrogels applied in MI treatment are systematically summarized (Table II). Additionally, quantitative therapeutic outcomes of various intelligent responsive hydrogels in MI treatment are presented (Table III). These comparative analyzes enable evidence-based evaluation of material selection, degradation profiles, mechanical compatibility, and therapeutic efficacy across different responsive platforms.

**TABLE II.** Characteristics and therapeutic effects of different responsive hydrogels for MI therapy.

Type of responsive hydrogel	Material or chemical bond	Load composition	Injection method	Degradation time	Mechanical properties	Crucial therapeutic effects on MI	References
pH-responsive hydrogel	Imine bonds, MSN-NH-TMA, PEGCHO, α-CD	miR-21-5p	Intramyocardial injection	28 days	G′ > G″	Immune modulation Angiogenesis	60
	Schiff base, OHA, Col-CDH, MWCNT	Metformin MSC-Exos	Intramyocardial injection	21 days	G′ > G″ conductivity (7.61 × 10^−4^ S/cm) Elastic modulus (38 kPa) (OHA/Col-CDH/MWCNT-0.2)	Restoration of electrical signal conduction Antioxidation Angiogenesis	^61^
	Borate ester bonds PVA, OSA, borax, tannic	MXene-TA nanozymes	Intramyocardial injection	————	G′ > G″ Conductivity (5.0 × 10^−4^ S/cm (PST0.4%) Elastic modulus > 40 kPa (PST6%) Self-healing	Reprogrammed metabolism activated NAD+-dependent pathways	^63^
ROS-responsive hydrogel	Diselenide bonds, HSe-CD, HA-CD, HA-Ad	miR-19a/b-cholesterol	Intramyocardial injection	>20 days	Shear-thinning behavior	Promotes cardiomyocyte proliferation Functional recovery	85
	Boronate ester Chitosan	NO	Intramyocardial injection	>21 days	G′ > G″ Shear-thinning behavior	Regulates ROS/NO balance	86
	mPEG-P (Met-co-Ala)	FTY720	Intramyocardial injection	35 days	G′ 490.0 Pa (6.0 wt. % PM)	Antioxidation Anti-apoptotic	^95^
MMP-responsive hydrogel	Collagen, GSH, Sulfo-SMCC, Amide bond	Recombinant protein GST-TIMP-bFGF	Intramyocardial injection	>8 days	G′ > G″	Angiogenesis, Improve heart function	124
	HA-SH, ALG-CHO, TA, MMP-2-cleavable peptides	DPCA	Intramyocardial injection	>6 days	G′ > G″ Soft mechanical property (200–300 Pa) Shear-thinning behavior Conductivity (8.8 × 10^−5^ S/cm)	Stabilize HIF-1α Improve heart function	^126^
Thermoresponsive hydrogel	PNIPAAm, SWCNTs	BASCs	Intramyocardial injection	————	Conductivity	Enhance cell adhesion and proliferation	140
	Pluronic F127, PEDOT:PSS, PDA	α-TOH	Surface of the infarct area	————	G′ > G″ conductivity (1.5 S/m) High stretchability (>140%) Low elastic modulus (>35 kPa) Young's modulus (80 kPa)	Modulate the post-MI infarct microenvironment Enhances cardiac repair Improve heart function	^145^
pH/thermo-Responsive hydrogel	P(CS–CA–NIPAM)	OSM	Intramyocardial injection	>30 days	G′ > G″	Stimulate cell proliferation Angiogenesis Improve heart function	164
pH/ROS-responsive hydrogel	Imine bonds Borate ester bonds OXP, GD	PDA-RA NPs	Intramyocardial injection	>8 days	G′ > G″ (OGPDR) Shear-thinning behavior Conductivity (6.27 × 10^−4^ S/cm) Elastic modulus (45.35 kPa)	Improve heart function Promotes repair	28

**TABLE III.** Quantitative comparison of therapeutic outcomes among different stimuli-responsive hydrogel systems. Data are presented as percentage reduction or improvement relative to MI control groups, or as fold change (angiogenesis markers) compared to controls, as reported in the original studies. “————” indicates data not reported in the original publication. Myocardial infarct size was assessed by triphenyltetrazolium chloride (TTC) staining. Left ventricular ejection fraction (LVEF) was evaluated by echocardiography at 4 weeks post-treatment. Angiogenesis was quantified by immunohistochemical staining of endothelial and vascular smooth muscle cell markers, including CD31^+^ (endothelial cells), α-SMA^+^ (vascular smooth muscle cells), and vWF^+^ (von Willebrand factor).

Hydrogel type	Responsive mechanism	Animal model	Infarct size reduction (%)	LVEF improvement (%)	Angiogenesis	References
Gel@MSN/miR-21-5p	pH-responsive	Mini pig MI	About 20%	About 10%	Vascular volume +50 mm^3^ α-SMA^+^ density (fold) +2.0 CD31^+^ density (fold) +1.8	60
OHA/Col-CDH/MWCTN-Met-EXO hydrogel	pH-responsive	Rat MIRI	About 50%	About 25%	α-SMA^+^ density (fold) +2.4 vWF^+^ density (fold) +2.5 CD31^+^ density (fold) +1.1	61
HSe-CD-HA-CD-HA-Ad-miR-19a/b hydrogel	ROS-responsive	Mini pig Rat MI	About 22% About 12%	About 28% About 34%	CD31^+^/α-SMA^+^ vessel density↑	85
S1P/SS-31/lipo-loaded hydrogel	ROS-responsive	Rat MI	About 42%	About 24%	α-SMA^+^ density (fold) +1.8 vWF^+^ density (fold) +1.5	91
GST-TIMP-bFGF/collagen-GSH hydrogel	MMP-responsive	Rat MI	————	About 20%	α-SMA^+^ density (fold) +1.1 vWF^+^ density (fold) +2.2 CD31^+^ density (fold) +2.0	124
FPDA hydrogel	Thermoresponsive	Rabbit MI	————	About 12%	α-SMA^+^ density (fold) +4.0 CD31^+^ density (fold) +2.1	145
OGDPR hydrogel	pH/ROS-responsive	Rat MI	About 32%	About 40%	α-SMA^+^ density (fold) +2.5 vWF^+^ density (fold) +2.5	28
FGPP@AST hydrogel	pH/thermo-responsive	Rat MI	About 12%	About 30%	α-SMA^+^ density (fold) +6.8 vWF^+^ density (fold) +5.7	62

However, current studies still show clear limitations. Most designs focus on simple dual-stimuli systems, neglecting other key pathological cues such as elevated MMP levels and hypoxia. Many platforms also lack hierarchical responsiveness, failing to align stimulus activation with the dynamic stages of MI from inflammation to proliferation and remodeling. Evidence to date does not identify a single optimal responsive mechanism for MI therapy, as variations in models, protocols, and evaluation timelines make comparisons difficult. Future work should emphasize standardized head-to-head studies with unified criteria and stage-matched interventions to clarify which mechanisms or combinations yield the best outcomes. Researchers should also aim to establish integrated multi or quadruple responsive frameworks sensitive to pH, ROS, MMPs, and hypoxia, supported by computational modeling to predict synergistic effects and guide rational design. Incorporating feedback regulation will allow therapeutics to dynamically modulate subsequent responses. Before clinical translation, careful assessment is needed to determine whether added system complexity delivers tangible therapeutic benefits in large animal models.

### F. Stage-specific MI microenvironmental characteristics and matched responsive strategies

The pathophysiological evolution of MI follows a temporal sequence comprising three overlapping yet distinct stages: the inflammatory phase, the proliferative phase, and the remodeling phase.^167^ Each stage is characterized by specific microenvironmental alterations, which provide unique opportunities for the intervention of stimulus-responsive hydrogels (Table IV).

**TABLE IV.** Temporal framework of MI pathophysiology and optimal responsive hydrogel strategies.

MI phase	Timeline	Dominant microenvironmental characteristics	Optimal responsive mechanisms	Representative systems	Key therapeutic outcomes
Inflammatory phase	0–7 days	Explosive ROS generation Inflammatory cell infiltration Severe tissue acidosis	pH-responsive ROS-responsive Thermoresponsive	Gel@VHH^96^ Col@Gel-colchicine^143^	Attenuate myocardial injury Anti-inflammation Anti-oxidation
Proliferative phase	7–28 days	Macrophage polarization Fibroblast proliferation Peak MMP-2/9 expression Sustained oxidative stress Active angiogenesis	MMP-responsive ROS/MMP-responsive pH/MMP-responsive	MPGC4^125^ HSD/DFO@GMs^38^	Regulate macrophage polarization Promote angiogenesis Suppress oxidative stress
Remodeling phase	28 days-months	Scar tissue maturation Collagen cross-linking Chronic oxidative stress Electrical decoupling	Multi-stimuli responsive	OGDPR^28^ S1&FT/Lipo-QCFT^91^	Anti-fibrosis Suppress oxidative stress Inhibit myocardial remodeling Improves heart function

During the early pathophysiological progression of MI, the inflammatory phase emerges and is characterized by severe ischemia-induced cardiomyocyte death, which subsequently triggers extensive inflammatory cell infiltration and oxidative burst.^168,169^ The microenvironment becomes profoundly acidotic owing to anaerobic metabolism, excessive ROS generation, and localized hyperthermia.^170^ During this critical window, ROS-responsive, pH-responsive, and thermoresponsive hydrogels loaded with antioxidants or anti-inflammatory agents can provide rapid therapeutic intervention, effectively mitigating oxidative damage and suppressing excessive inflammatory responses. In the proliferative phase, macrophages undergo phenotypic transition from M1 to M2, while fibroblasts proliferate and differentiate into myofibroblasts to facilitate tissue repair.^171,172^ This phase is marked by active angiogenesis, tissue consolidation, and granulation tissue formation with organized collagen deposition.^173^ The microenvironment is defined by elevated MMP-2/9 expression, persistent oxidative stress, and gradual pH normalization. At this stage, MMP-responsive hydrogels and dual-responsive systems (ROS/MMP or pH/MMP) are particularly suitable for targeted intervention, enabling controlled release of pro-angiogenic factors and immunomodulatory agents to support constructive tissue remodeling while suppressing excessive inflammatory responses. The remodeling phase is characterized by scar maturation, in which type I collagen replaces type III collagen, resulting in significantly increased tissue stiffness, reduced cellular density accompanied by fibroblast apoptosis, stabilization and partial regression of the vascular network, chronic redistribution of mechanical stress, and potential progression toward adverse ventricular remodeling that may lead to heart failure.^174^ Multi-stimuli-responsive hydrogels offer sustained and controlled release profiles to address the complex and evolving microenvironment, preventing adverse ventricular remodeling and supporting long-term functional recovery. These systems can simultaneously respond to residual ROS, subtle pH fluctuations, and sustained matrix metalloproteinase activity, thereby delivering anti-fibrotic agents, improving electrical conduction, and promoting favorable ventricular remodeling throughout extended therapeutic windows.

The responsive release kinetics of hydrogels should be highly consistent with the pathological progression of MI. pH-responsive hydrogels can sense the acidic microenvironment, with degradation rates dependent on the type of chemical bonds. For instance, imine bonds typically exhibit a degradation half-life (t_1_/_2_) of 1–3 h at pH < 5, and within a certain range, the reaction rate positively correlates with the pH value, thereby enabling rapid drug release. In ROS-responsive hydrogels, diselenide demonstrates rapid oxidation characteristics with degradation occurring within minutes to hours, while thioether bonds undergo relatively slower cleavage reactions, with reaction rates positively correlating with local ROS concentrations. In contrast, the degradation rate of MMP-responsive hydrogels primarily depends on local MMP-2/9 expression levels, typically requiring 3–7 days for significant degradation and drug release in MI treatment. Temperature-responsive hydrogels, relying on physical phase transitions, can achieve nearly instantaneous responses, representing the fastest response among all stimuli-responsive types, albeit with limited specificity. Therefore, during the acute phase of myocardial infarction, where the infarct microenvironment is characterized by severe oxidative stress and low pH conditions, ROS- and pH-responsive hydrogels demonstrate greater advantages due to their degradation kinetics. Conversely, during the proliferative repair phase, MMP-responsive hydrogels are more suitable. Only by precisely matching the degradation kinetics of various responsive hydrogels with different pathological stages of MI can optimal therapeutic outcomes be achieved.

## III. ADVANTAGES AND CHALLENGES OF INTELLIGENT RESPONSIVE HYDROGELS

With the rapid advancement of materials science and biotechnology, intelligent responsive hydrogels have demonstrated remarkable potential in the treatment of MI. These materials can dynamically respond to pathological microenvironments, enabling on-demand release of therapeutic agents. Their precise modulation capabilities open new avenues for MI therapy. Despite encouraging research progress, the development of intelligent responsive hydrogels still faces significant challenges. Future efforts should focus on continuous innovation to develop diversified, intelligent responsive materials and to integrate them with emerging therapeutic strategies, with the ultimate goal of providing more effective and precise treatment options for patients with MI.

### A. Advantages of intelligent responsive hydrogels

#### 1. Spatiotemporally controlled drug release

By incorporating environmentally sensitive moieties or polymers into hydrogel structures, engineered intelligent responsive hydrogels exhibit specific responsiveness to the pathological microenvironment of MI, surpassing the capabilities of conventional hydrogels. This feature enables spatiotemporally controlled, dose-dependent release of drugs or therapeutic factors at the lesion site, effectively increasing local drug concentrations while significantly reducing systemic side effects. Furthermore, hydrogels can be designed to target distinct pathological phases of MI, including the inflammatory, proliferative, and maturation phases, thereby delivering stage-specific therapeutic effects to enhance treatment efficacy. For instance, Hu *et al.* constructed a MI-responsive hydrogel by forming boronate ester linkages between the 3-aminophenylboronic acid (BA) groups of CMC-BA and the dihydroxyl groups of polyvinyl alcohol (PVA).^175^ The hydrogel was further loaded with curcumin-encapsulated PLGA nanoparticles (PLGA@Cur NPs) and recombinant human collagen type III (rhCol III). This multifunctional hydrogel degraded under acidic and high ROS conditions, enabling on-demand release of Cur and rhCol III. *In vitro* results showed that at pH 5.0 and 0.2 mM H_2_O_2_, the cumulative release of Cur and rhCol III reached around 80% and 60% by day 8, respectively, due to the hydrogel's dual responsiveness. During the acute inflammatory phase of MI, rapid Cur release suppressed early oxidative damage and inflammation, while sustained rhCol III release supported later cell proliferation and angiogenesis.

#### 2. Excellent biocompatibility and inherent safety

The clinical translation potential of intelligent responsive hydrogels for MI therapy is critically dependent on their biocompatibility, a property that governs the safety of dynamic interactions between materials and biological systems at the molecular, cellular, and systemic levels.^176,177^ Biocompatible materials are defined by their ability to interact with biological entities, including cells, tissues, and immune systems, without eliciting toxicity, inflammation, or immune rejection in specific applications.^178^ Intelligent responsive hydrogels are typically fabricated from biocompatible materials such as natural polysaccharide-derived hydrogels. These hydrogels exhibit molecular structures that closely resemble those of the ECM, demonstrating high affinity and low immunogenicity, thereby ensuring their safe and effective application in MI treatment.^179,180^ For example, Jiang *et al.* designed a responsive injectable hydrogel [P(CS-CA-NIPAM)].^164^ CCK-8 and live/dead staining showed H9C2 cell viability above 90%, indicating negligible cytotoxicity of the polymer. Calcein-AM/PI staining further confirmed the excellent biocompatibility of the material, as almost no cell death was observed in H9C2 cells.

Intelligent responsive hydrogels generally exhibit favorable biodegradability, enabling safe *in vivo* degradation and effectively mitigating long-term retention risks.^181,182^ Their degradation process is temporally synchronized with the myocardial repair cycle. Prior to clinical application, intelligent responsive hydrogels must undergo rigorous biocompatibility and safety assessments. Evaluation methodologies include cytotoxicity assays, such as the MTT assay or lactate dehydrogenase (LDH) release assay, to assess toxicity to cardiomyocytes or fibroblasts, and Hematoxylin-Eosin (H&E) staining to evaluate potential damage to visceral cells, including hepatic, splenic, pulmonary, and renal tissues.^183^ These stringent evaluations ensure the safety and efficacy of intelligent responsive hydrogels for clinical use.

#### 3. Multifunctional integration and synergistic therapy

Multifunctional integration and synergistic therapy have emerged as the core development direction for intelligent responsive hydrogels, aiming to achieve efficient and precise drug delivery while promoting tissue repair and regeneration through multiple functional modules. Specifically, therapeutic agents or nanoparticles can be encapsulated within hydrogels, with drug release triggered by environmental stimuli to enable targeted therapy.^184,185^ Furthermore, bioactive factors such as growth factors and cytokines can also be incorporated to enhance cell proliferation and migration, thereby accelerating tissue repair.^186,187^ Concurrently, hydrogels with biocompatibility and biodegradability can serve as scaffolds for cell delivery, providing robust support for tissue regeneration and repair.^188^ Innovatively, research is exploring the integration of biosensors into hydrogels to enable real-time monitoring of physiological parameters, offering critical insights for disease diagnosis and treatment. This direction undoubtedly opens new frontiers for future research.

### B. Challenges of intelligent responsive hydrogels

#### 1. Complexity in material design and fabrication

Intelligent responsive hydrogels face significant clinical translation hurdles due to complex chemical synthesis and modification, leading to difficult fabrication and high costs. From a material design perspective, key performance indicators must be satisfied: excellent biocompatibility to prevent immune rejection, mechanical properties matching cardiac tissue to provide optimal support, and molecular features responsive to specific stimuli for environmental adaptability.^189,190^

To achieve multifunctionality in intelligent hydrogels, multicomponent systems typically require the integration of polymers, bioactive substances, and nanoparticles. However, the nonuniform distribution of these components within the hydrogel matrix can lead to inconsistent material properties, thereby compromising therapeutic efficacy. Furthermore, microstructural parameters such as pore size and porosity significantly influence cell migration, nutrient transport, and tissue regeneration.^191^ Consequently, precise control over the microstructure necessitates optimization of fabrication methodologies. Notably, large-scale production encounters critical technical hurdles in ensuring batch-to-batch consistency and stability.^192,193^ This challenge underscores the need to establish stringent quality control systems with real-time monitoring throughout all manufacturing stages. Additionally, appropriate storage conditions, including cryopreservation, controlled humidity, and protection from light, are essential for preserving the integrity of the three-dimensional hydrogel network and preventing structural denaturation.^194^

In addition, understanding structure–activity relationships (SARs) is fundamental to rational hydrogel design. Building on the representative intelligent responsive hydrogels discussed in the previous sections, several key structure–activity relationships can be identified. First, chemical bond stability plays a crucial role, as lower bond dissociation energy not only provides higher sensitivity to external stimuli but also increases the risk of premature degradation.^195,196^ Second, cross-linking density should be optimized so that the elastic modulus matches the stiffness of cardiac tissue, balancing mechanical support with cellular infiltration.^197^ Third, maintaining a proper balance between hydrophilic and hydrophobic components is essential for responsiveness and structural stability.^198^ Fourth, conductive filler loading must be carefully controlled to achieve adequate conductivity while preserving biocompatibility.^199^ Looking ahead, future studies should aim to develop quantitative structure–activity relationship models that link structural parameters with therapeutic outcomes, enabling predictive material design.

#### 2. Limitations in sensitivity and controllability of intelligent responsive systems

The responsive behavior of intelligent hydrogels typically relies on bond cleavage or material responses triggered by specific chemical signals, exhibiting distinct threshold characteristics.^200^ Significant hydrogel responses occur only when the stimulus intensity exceeds a critical threshold; otherwise, incomplete bond cleavage may compromise therapeutic efficacy.^201^ For pH-responsive hydrogels, the typical activation condition occurs at pH values below 7. However, variations in patients' infarct size, metabolic status, or local blood supply may cause pH fluctuations beyond the trigger range, leading to premature degradation or delayed drug release, thereby impairing therapeutic outcomes. Moreover, physiological states and microenvironmental heterogeneity among individuals further modulate hydrogel responsiveness. Enzyme-responsive hydrogels depend on specific enzyme expression levels; however, patient age, genetic background, or medication use may alter enzymatic activity, thereby affecting drug release profiles. Finally, most intelligent hydrogels display single-phase release behavior, limiting their ability to dynamically adapt to disease progression. For instance, thermoresponsive hydrogels release drugs at physiological temperatures, but sustained fever in patients can exhaust the material's phase-transition capacity, resulting in reduced therapeutic efficacy.

The optimization of sensitivity and responsive performance in intelligent responsive hydrogels fundamentally represents the challenge of achieving compatibility between the material design and complex pathophysiological microenvironments, posing new difficulties for researchers. Future studies should focus on further refining hydrogel materials, constructing multimodal cooperative response systems, mimicking the hierarchical structures of biological tissues, and optimizing response mechanisms. An emerging research frontier involves leveraging artificial intelligence to predict individual patient parameters, such as enzymatic activity and pH fluctuations, in order to tailor hydrogel activation thresholds for personalized therapeutic applications.

#### 3. Clinical translation barriers

**FIG. 9.**
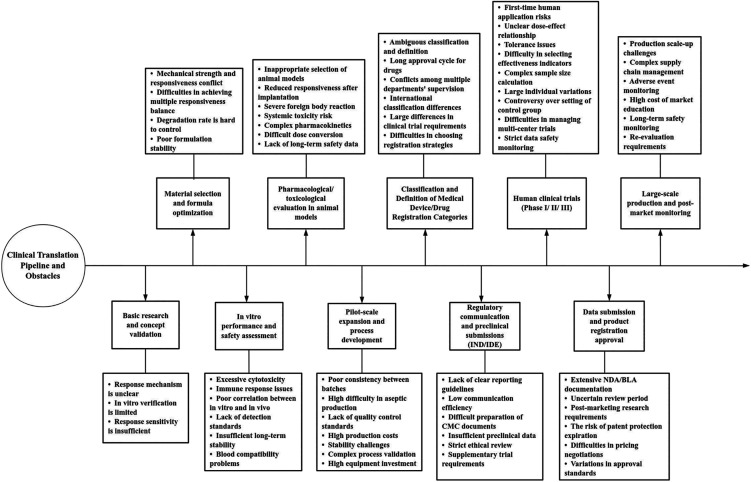
Clinical translation pathways and bottlenecks of intelligent responsive hydrogels.

Despite significant research breakthroughs in the hydrogel field, intelligent responsive hydrogels continue to encounter substantial bottlenecks in clinical translation (Fig. 9).

One major obstacle to clinical translation is that hydrogels showing promising results *in vitro* often fail to reproduce the same therapeutic outcomes *in vivo*. This inconsistency stems from several fundamental limitations. The pathological process of MI is inherently complex, involving multiple cell types, dynamic microenvironmental changes, and systemic regulation, whereas simplified *in vitro* models cannot fully mimic these conditions.^202^ Moreover, static culture systems fail to replicate the cyclic mechanical stress generated by the beating heart, leading to mechanical fatigue, shear-induced drug leakage, and delamination at the implant-tissue interface. In addition, pharmacokinetic factors such as systemic clearance, vascular washout, and protein binding reduce local drug availability.^203^ Finally, immune activation, foreign body reactions, and chronic inflammation *in vivo* can all diminish therapeutic efficacy, despite favorable biocompatibility results in preliminary tests.^204,205^ To bridge this translational gap, future studies should prioritize the development of dynamic *in vitro* models that mimic temporal fluctuations in pH, ROS, and MMP levels as well as mechanical loading. Advanced imaging modalities such as Magnetic Resonance Imaging (MRI) and Positron Emission Tomography (PET) are needed to track hydrogel distribution, degradation, and drug release in real time. Furthermore, the identification of relevant biomarkers will be essential to recognize early predictors of *in vivo* failure during *in vitro* screening.

In addition, in order to achieve true clinical translation, patient stratification is essential, requiring comprehensive consideration of the pathological stage, clinical characteristics, and hydrogel properties to enable precise and personalized therapy. It is essential to evaluate strategies that maximize therapeutic efficacy while minimizing potential risks, aligning hydrogel functionalities with the individualized pathological needs of patients. According to the pathophysiological progression of myocardial infarction, appropriate responsive hydrogels were matched to each disease stage (Table IV) to meet stage-specific therapeutic requirements and achieve precise drug release. However, it is worth noting that during the acute phase of myocardial infarction, when hemodynamic instability may occur, intramyocardial injection should be used with caution. In such cases, catheter-based delivery may represent a safer and more feasible alternative. Additionally, patient stratification based on the infarct size and location is also crucial. For anterior wall myocardial infarction, priority may be given to mechanically supportive intelligent responsive hydrogels, which can provide maximal structural reinforcement to prevent cardiac rupture and limit ventricular remodeling. In contrast, for inferior wall myocardial infarction, intelligent responsive hydrogels designed to modulate fluid balance and electrical conduction may be more appropriate. Finally, a comprehensive assessment of the patient's overall clinical profile is paramount. Age, comorbidities, and revascularization status must inform hydrogel selection. For younger patients, intelligent responsive hydrogels with enhanced bioactivity that promote long-term repair and tissue regeneration may be preferable. In contrast, for elderly patients or those with multiple comorbidities, mechanically supportive hydrogels with high safety and low immunogenicity are more suitable. Moreover, in patients with diabetes or impaired renal function, hydrogel components that rely on renal metabolism should be avoided to minimize potential systemic risks. Future research should focus on developing an evidence-based stratification framework and validating it through prospective clinical studies, ultimately achieving truly individualized therapy for MI.

Several fundamental barriers also continue to impede clinical translation. First, long-term safety assessment of intelligent responsive hydrogels remains significantly inadequate. Although these hydrogels exhibit favorable *in vivo* biocompatibility, the long-term immunogenicity and toxicity of their degradation products or loaded nanomaterials remain poorly characterized. Current studies are largely restricted to short-term observations, leaving long-term effects insufficiently evaluated. Therefore, establishing a standardized safety evaluation framework will require systematic animal model studies and multicenter clinical trials. Second, the clinical research infrastructure remains underdeveloped. As an emerging class of biomaterials, intelligent responsive hydrogels are still primarily investigated in preclinical animal studies, with extremely limited clinical research. Consequently, their therapeutic efficacy cannot be directly extrapolated to human outcomes. Clinical patients, often elderly individuals with comorbidities such as diabetes or hypertension, present more complex physiological microenvironments, placing greater demands on smart responsive hydrogels. Furthermore, the absence of unified clinical trial design protocols and efficacy evaluation criteria hinders cross-study data comparison and integration, severely impeding clinical translation. Thus, developing standardized clinical research frameworks has become pivotal for advancing this field. Finally, in the context of MI therapy, although endocardial injection has emerged as a major research focus due to its precision, this technique requires considerable operator expertise and carries the risk of severe complications, including myocardial perforation and arrhythmias. A critical technical challenge persists: ensuring injection precision while mitigating procedural risks through the development of safer and more effective delivery technologies.

## IV. CONCLUSION

Intelligent microenvironment-responsive hydrogels offer precise and adaptive therapeutic strategies for MI by responding to pathological cues such as pH, ROS, temperature, and enzyme activity. These hydrogels enable controlled drug release, modulate inflammation, and promote tissue regeneration, showing great promise in targeted MI therapy. However, complex fabrication, high production costs, and limited clinical data hinder their translation. Future research should emphasize material optimization, biosafety evaluation, and artificial intelligence-guided design to achieve personalized and precise treatment. With continued interdisciplinary innovation, intelligent responsive hydrogels are poised to transform cardiac repair and advance regenerative cardiovascular medicine.

## Data Availability

The data that support the findings of this study are available from the corresponding authors upon reasonable request.
